# Radiologist-guided multimodal fusion network for differentiating parotid tumors on 3D non-contrast CT

**DOI:** 10.3389/fonc.2026.1926379

**Published:** 2026-10-15

**Authors:** Qin Yang, Wenjun Liao, Jianda Sun, Dacheng Mu, Minyang Yang, Qiaohua Li, Yue Zhao, Shichuan Zhang

**Affiliations:** 1 Department of Radiation Oncology, Precision Radiation in Oncology Key Laboratory of Sichuan Province, Sichuan Clinical Research Center for Cancer, Sichuan Cancer Hospital & Institute, Sichuan Cancer Center, University of Electronic Science and Technology of China, Chengdu, China; 2 Department of Radiation Oncology, Radiation Oncology Key Laboratory of Sichuan Province, Sichuan Clinical Research Center for Cancer, Sichuan Cancer Hospital & Institute, Sichuan Cancer Center, University of Electronic Science and Technology of China, Chengdu, China; 3 Department of Oncology, Meizhou People’s Hospital, Meizhou, China; 4 Department of Oncology, Bazhong City Hospital of Traditional Chinese Medicine, Bazhong, China

**Keywords:** expert knowledge-based attention, interpretability, multimodal fusion, non-contrast CT, parotid tumors

## Abstract

**Objectives:**

To improve the diagnostic accuracy of benign versus malignant parotid tumors using non-contrast CT. We propose a clinician-in-the-loop hybrid framework that mitigates low soft-tissue contrast by fusing semi-automated 3D visual features with expert semantic descriptors.

**Methods:**

This multi-center retrospective study analyzed non-contrast CTs from 367 patients. We developed AttenNet, a semi-automated model. First, an nnU-Net module automates 3D tumor segmentation, eliminating manual bounding boxes. A 3D-ResNet backbone then extracts volumetric features, which are dynamically fused with radiologist-provided semantic labels via a Multi-Head Cross-Attention Fusion (MHAF) mechanism. This allows the network to query visual morphology based on expert priors. Diagnostic performance and Gradient-weighted Class Activation Mapping (Grad-CAM) interpretability were evaluated.

**Results:**

The segmentation achieved a median Dice of 0.827. AttenNet (3D-ResNet) yielded an AUC of 0.908, significantly outperforming the image-only 3D-ResNet (AUC: 0.741) and expert-feature models (AUC: 0.821). External validation (*n* = 20) reached an AUC of 0.560–0.910.However, given the extremely small sample size, these results must be interpreted strictly as an exploratory proof-of-concept rather than definitive confirmation of generalizability. Grad-CAM confirmed that AttenNet highlights clinically essential geometric indicators-specifically tumor margins and internal heterogeneity-for differentiating malignancy, whereas single-modality baselines focused on background artifacts.

**Conclusion:**

This clinician-in-the-loop paradigm successfully integrates spatial imaging with expert reasoning. While multimodal fusion drove the primary performance gains, the MHAF mechanism provided enhanced spatial interpretability.

## 1 Introduction

Parotid gland neoplasms encompass both benign and malignant varieties (1, 2). Malignant tumors typically present in patients over 60 years of age with an equal gender distribution, whereas benign lesions are more prevalent in females (3). Given that precise preoperative diagnosis is paramount, noninvasive methodologies are essential when contrast-enhanced imaging or biopsy are contraindicated or unfeasible. Existing diagnostic modalities, including Computed Tomography (CT), Magnetic Resonance Imaging (MRI), Fine-Needle Aspiration (FNA), and frozen section analysis (4), are associated with specific limitations: MRI is limited by high costs (4); contrast-enhanced CT carries risks of nephropathy and allergic reactions (4); FNA is susceptible to sampling bias (5, 6); and non-contrast CT, despite its accessibility, offers limited diagnostic utility due to overlapping radiological features (7). This inherent accessibility positions non-contrast CT as an ideal candidate for Artificial Intelligence (AI) integration, leveraging deep learning algorithms to discern subtle patterns, resolve diagnostic ambiguity, and transform it into a precise instrument for clinical evaluation.

In recent years, AI has advanced rapidly in medical diagnosis. However, previous literature regarding parotid gland tumors has largely focused on multiparametric MRI, which evaluates entirely different physical modalities and sequences (8, 9). For non-contrast CT, where soft-tissue differences are inherently subtle, deep learning models are particularly valuable but face distinct challenges. Earlier classification models predominantly utilized 2D slices, a method that inevitably compromises the volumetric continuity and spatial geometric priors of the tumors, ultimately reducing model performance. While recent studies have transitioned to 3D convolutional networks to capture these critical spatial attributes, many still rely heavily on manual Region of Interest (ROI) delineation by radiologists to crop the tumor area. This manual intervention not only introduces subjective bias and workflow bottlenecks but also falls short of an automated image-processing pipeline (10, 11). Furthermore, extracting morphological features solely from a single modality, even in 3D, often hits a performance ceiling due to the limited anatomical contrast of plain CT scans (12, 13).Furthermore, the diagnostic efficacy of downstream classification models is inherently tied to the robustness of the upstream tumor localization. For instance, recent pioneering developments have introduced anatomy-aware frameworks designed specifically for the complex spatial constraints of parotid tumors, demonstrating exceptional segmentation performance on multimodal MRI (14). Integrating such dedicated, anatomy-aware segmentation modules into our human-in-the-loop classification framework represents a highly promising avenue for future research, potentially further refining the spatial inputs and mitigating localization-induced performance degradation.

Therefore, the primary objective of this study is to significantly improve the diagnostic accuracy of benign versus malignant parotid tumors, utilizing multimodal fusion as a strategic mechanism to overcome the physical limitations of non-contrast CT. To achieve this, we propose AttenNet, a novel clinician-in-the-loop hybrid framework. Our workflow addresses previous limitations through a two-stage sequential process: first, an upstream nnU-Net (15)module performs automated 3D tumor segmentation and spatial localization, completely eliminating the need for manual cropping by radiologists. Second, rather than relying solely on imaging data, the framework introduces explicit anatomical priors (e.g., shape, margin, and necrosis) provided by clinical experts. These structured semantic descriptors are dynamically integrated with 3D volumetric visual features using a Multi-Head Cross-Attention Fusion (MHAF) mechanism, allowing the imaging network to actively query relevant clinical attributes. Ultimately, to bridge the gap between algorithmic prediction and clinical trust, we employ pixel-level attribution mapping via Grad-CAM to visualize the model’s focus. By demonstrating that our attention-based fusion accurately grounds its diagnostic predictions on critical tumor margins and spatial geometries, this study provides a highly accurate, interpretable, and non-invasive alternative for parotid tumor evaluation.

## 2 Materials and methods

### 2.1 Data acquisition

This retrospective study included 367 patients with pathologically confirmed parotid tumors (260 benign, 107 malignant) from three tertiary hospitals (2008–2024). All participants underwent preoperative non-contrast CT (120 kVp, 1.0–1.25 mm slice thickness). Stratified 5-fold cross-validation was used for 347 patients from Centers A and B, reserving 20 patients from Center C for external validation (**Figure 1**; **Tables 1**, **2**). The external cohort (Center C) was assembled using the exact same stringent inclusion and exclusion criteria applied to the internal development cohorts (Centers A and B), as outlined in **Figure 1**.

**Figure 1:**
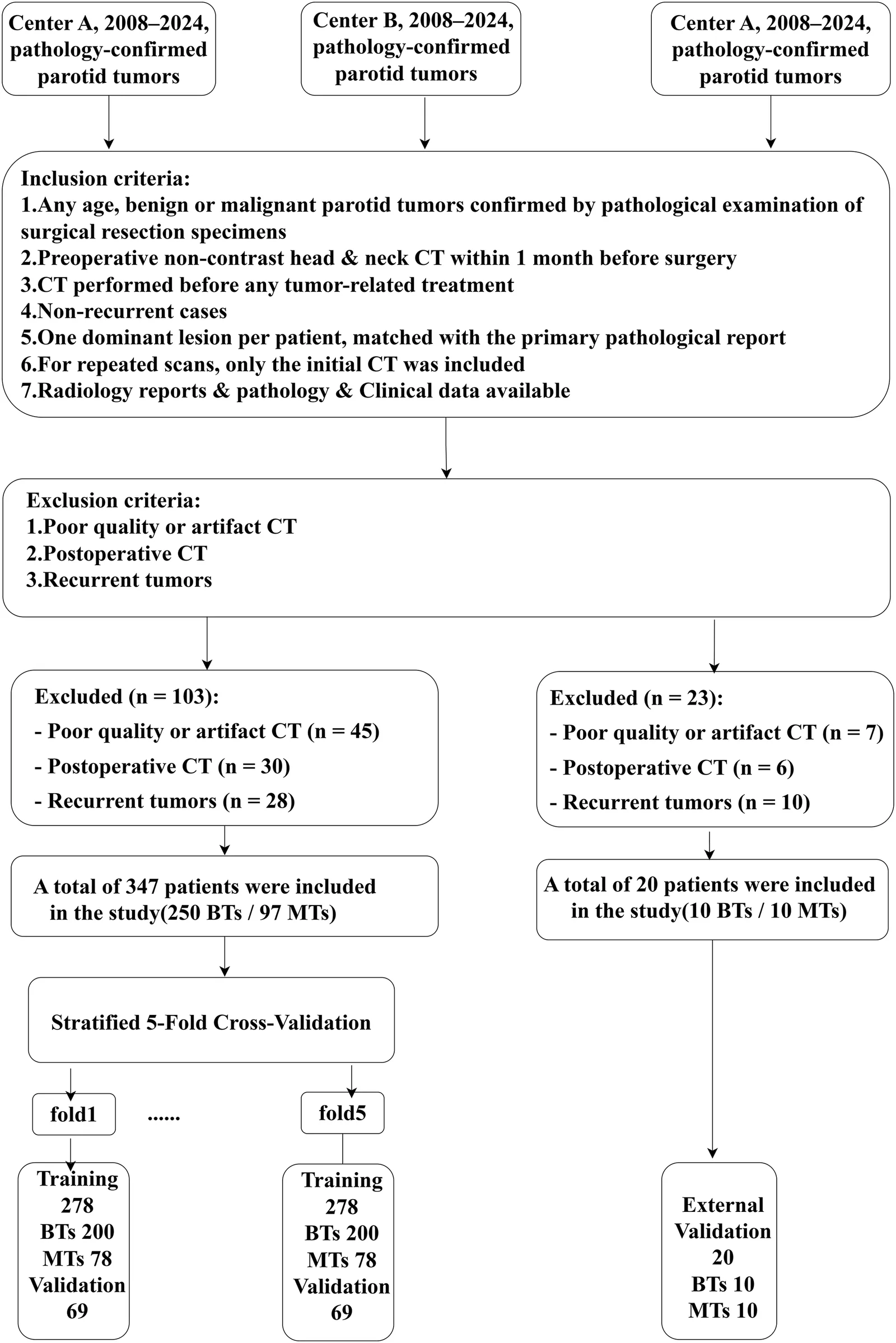
Flowchart of patient recruitment. From three centers, 347 patients (250 benign [BT], 97 malignant [MT]) were utilized for model development via stratified 5-fold cross-validation. An independent cohort of 20 patients (10 BT, 10 MT) was reserved for external validation.

**Table 1:** CT acquisition parameters across centers.

Center	Manufacturer	Tube voltage (kVp)	Tube current (mA)	Slice thickness (mm)	Reconstruction algorithm	Matrix Size
A	**GE**	**120**	**200–350**	**1.0**	**ASIR**	**512×512**
B	**Siemens**	**120**	**220–400**	**1.0**	**SAFIRE**	**512×512**
C	**Philips**	**120**	**150–300**	**1.25**	**iDose4**	**512×512**

*CT acquisition parameters across centers, acquisition parameters of non-contrast CT scans from the three participating centers. All scans were performed at 120 kVp with comparable matrix sizes (512 × 512) and thin-slice reconstructions (1.0–1.25 mm) using vendor-specific iterative reconstruction algorithms (ASIR, SAFIRE, and iDose4).

The bold values indicate the highest/best values to facilitate easier viewing and comparison.

**Table 2:** Distribution of parotid tumor cases across three hospitals.

Hospital	Cases (*n*)	BT *n* (%)	MT *n* (%)	Female *n* (%)	Male *n* (%)	Age(mean ± SD,years)
A	314	227(72.3)	87(27.7)	180(57.3)	134(42.7)	50.2 ± 6.1
B	33	23(69.7)	10(30.3)	15(45.5)	18(54.5)	49.6 ± 5.8
C	20	10(50.0)	10(50.0)	9(45.0)	11(55.5)	51.1 ± 5.5
Total	367	260(70.8)	107(29.2)	204(55.6)	163(44.4)	50.3 ± 6.0

*Data are expressed as number of patients with percentage in parentheses unless otherwise specified. Age is presented as mean ± standard deviation (SD).

### 2.2 Image processing

The dataset was anonymized and renamed in accordance with nnU-Net (15) requirements; processed information was summarized in four parts.

For the spatial and intensity preprocessing of the raw non-contrast-enhanced CT scans, we adhered to the standardized data pipeline established by the nnU-Net framework. Specifically, to address heterogeneous physical resolutions across different clinical centers, all CT volumes were automatically resampled to a unified target voxel spacing using third-order spline interpolation. For intensity normalization, the Hounsfield Unit (HU) values were clipped based on the empirical 0.5th and 99.5th percentiles of the foreground regions to exclude extreme outliers (e.g., metallic artifacts or air). Subsequently, global z-score standardization was applied by subtracting the mean and dividing by the standard deviation of the clipped foreground intensities. This entire preprocessing pipeline was executed programmatically without manual intervention.

Binary segmentation masks: In a blinded review conducted by three head-and-neck radiologists with over 10 years of experience, ROIs delineated on non-contrast CTs using 3D Slicer, along with preliminary benign/malignant assessments, were resolved via consensus to fine-tune nnU-Net; inter-observer variability in manual segmentation across 347 cases resulted in pairwise Dice Similarity Coefficients ranging from 0.86 to 0.89 (mean: 0.877; see **Supplementary Table S4**).

Expert visual guidance: Three experts, blinded to clinical and histopathological data, independently evaluated non-contrast CT visual features, resolving discrepancies via consensus. To prioritize clinical interpretability, semantic features were categorically encoded prior to model training: tumor shape (round/oval=0; irregular=1), margin (clear=0; indistinct=1), intratumoral necrosis (no=0; yes=1), and CT density (uniform=0; non-uniform=1) (**Table 3**). The average time required for semantic feature extraction by the three experts is provided in the **Supplementary Tables S1**-**2**. The semantic feature extraction required approximately 48.3 to 67 seconds per case, integrating efficiently into routine radiological review. This pre-encoding ensured independence from cross-validation folds. Inter-rater agreement was substantial to almost perfect, achieving Fleiss’ Kappa values between 0.74 and 0.85 (**Supplementary Tables S1**-**1**).Pathology-confirmed tumor categories: For supervised training, tumor categories confirmed by pathology were assigned binary labels (benign = 0, malignant = 1).

**Table 3:** Distribution of visual-semantic features between benign and malignant parotid tumors.

Visual-semantic features	Benign tumors (*n* = 260)	Malignant tumors (*n* = 107)
Tumor morphology, regular/irregular (0/1)	201/59	25/82
Tumor margin, well-defined/ill-defined (0/1)	216/44	17/90
Tumor necrosis, absent/present (0/1)	151/109	60/47
CT attenuation, homogeneous/heterogeneous (0/1)	121/139	20/87

*Data are presented as number of patients. Binary features were encoded as 0/1, where 0 = negative and 1 = positive.

To rigorously prevent evaluation circularity and ensure methodological transparency, the semantic feature extraction process was strictly compartmentalized across the development and external validation phases. For the internal development cohorts (Centers A and B), semantic features were extracted by the institutional radiologists, who were blinded to the pathological ground truth. In contrast, to evaluate the true generalizability of the human-in-the-loop framework, semantic features for the independent external cohort (Center C) were extracted by a separate, newly invited expert radiologist with no involvement in the model training phase or internal feature extraction. During the comparative evaluation, this independent external reader was provided only with non-contrast CT volumes and their basic demographics. The reader remained strictly blinded to the final pathology, model architecture, and internal training data.

## 3 Methods

### 3.1 Network structure

A deep learning framework can classify parotid tumors using CT images via segmentation and attention-based multimodal fusion (**Figure 2**).

**Figure 2:**
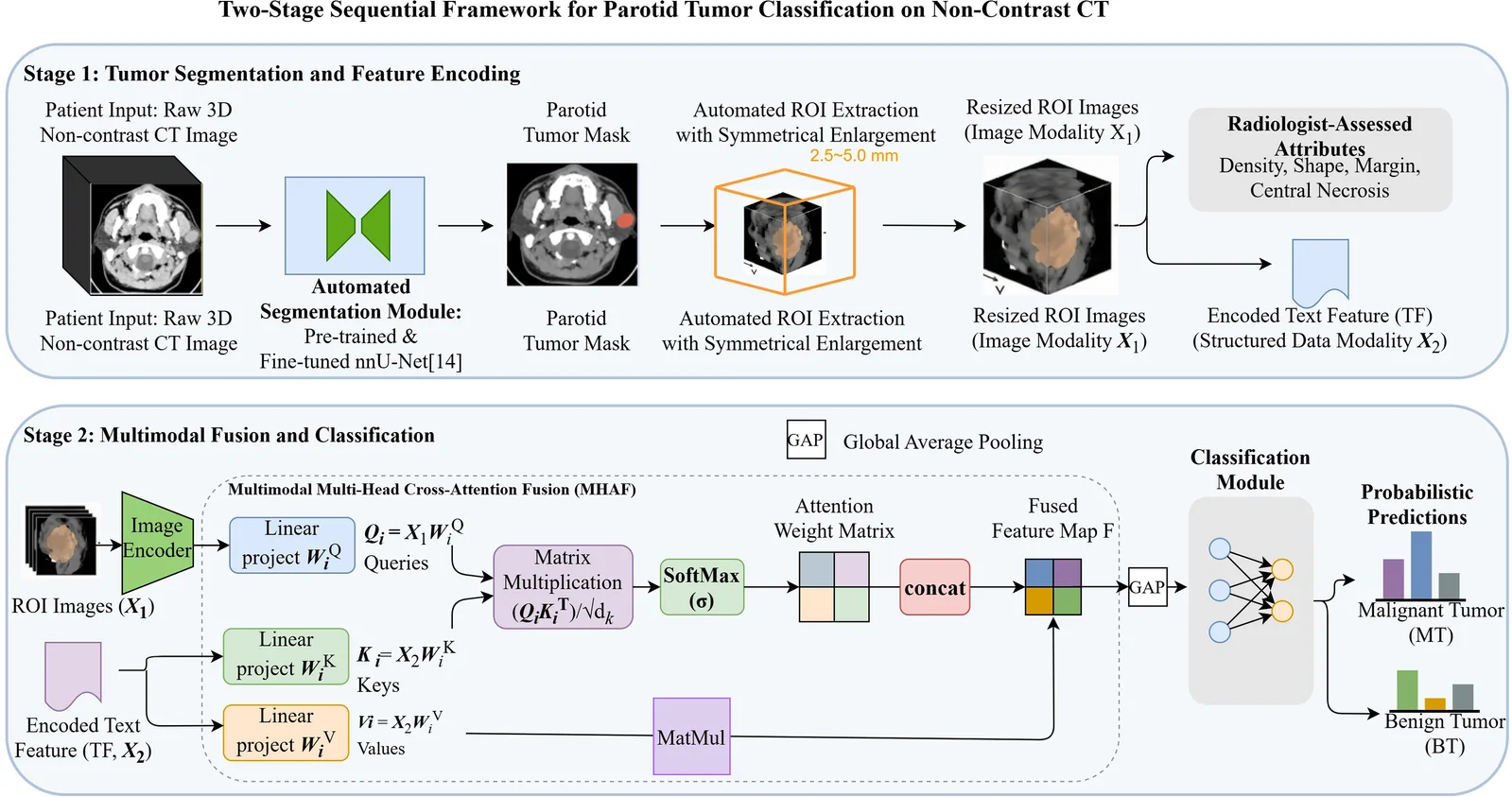
Stage 1: tumor segmentation and feature encoding; Stage 2: multimodal fusion and classification. Stage 1 uses nnU-Net for masks and ROI extraction, plus clinical text features. Stage 2 integrates imaging and text via MHAF, generating fused features for MT/BT prediction.

#### 3.1.1 Stage 1: Automated segmentation and feature encoding

As the primary objective of the segmentation module was to provide a standardized, coarse spatial localization for bounding box cropping rather than generalized pixel-wise diagnosis, the nnU-Net was trained on the internal development cohort. Consequently, the reported segmentation metrics reflect the model’s overall anatomical fitting capability across the dataset. Crucially, to minimize spatial feature leakage, the bounding box was symmetrically expanded by 2.5–5.0 mm to include peritumoral context, ensuring the downstream classifier did not rely solely on the exact segmentation boundaries.

##### 3.1.1.1 Tumor segmentation and ROI extraction

We employ a fine-tuned nnU-Net (14) to extract a binary mask $M\in {\left\{0,1\right\}}^{H\times W\times D}$from a CT volume $I\in R^{H\times W\times D}$. The bounding box derived from ***M*** is expanded by δ∈[2.5,5] mm along all spatial axes; subsequently, the region is cropped and resized to form a fixed-size tensor, $X_{1}\in R^{C\times H^{\prime }\times W^{\prime }\times D^{\prime }}$. The nnU-Net acts exclusively as a coarse spatial localizer. By forcing the classification network to independently extract morphological features from the raw crop rather than relying on pixel-wise prior probabilities from the segmentation mask, we maintain a strict functional boundary between localization and classification.

##### 3.1.1.2 Radiologist-assessed attribute embedding

We define the categorical attributes A={a1, a2, a3, a4} as density, shape, margin, and central necrosis. Each attribute is label-encoded and subsequently embedded via a learnable linear mapping:

$X_{2}=Linear\left(Embedding\left(A\right)\right)$ The projection of sparse categories into a latent space enables the model to learn semantic correlations between clinical descriptors through the use of the structured vector $X_{2}\in R^{1\times d}$.

##### 3.1.1.3 Image feature extraction backbones

For the extraction of volumetric visual features from the non-contrast CT ROI ( $X_{1}\in R^{C\times H^{\prime }\times W^{\prime }\times D^{\prime }}$), we evaluated two distinct 3D convolutional architectures as the backbone networks: 3D-ResNet and 3D-EfficientNet.

3D-ResNet is characterized by its use of residual connections. This architectural design creates shortcut paths for gradient flow, effectively mitigating the vanishing gradient problem inherent in training deep neural networks. Consequently, 3D-ResNet can be constructed with deeper layers to capture highly abstract, multi-scale spatial textures and deep semantic features of the tumor volumes.

In contrast, 3D-EfficientNet employs a compound scaling methodology. Rather than arbitrarily increasing a single dimension like network depth, it systematically balances network depth, width (number of channels), and input resolution using a fixed set of optimal scaling coefficients. This design enables 3D-EfficientNet to extract robust and fine-grained 3D features with significantly optimized computational efficiency.

#### 3.1.2 Stage 2: Multimodal multi-head cross-attention fusion

To effectively model the complex inter-modality dependencies between visual cues and clinical descriptors, we propose a MHAF module based on a cross-attention mechanism. Rather than simply concatenating feature vectors, MHAF uses the high-dimensional spatial feature maps extracted by the 3D-ResNet backbone as Queries (Q) to interact with the distilled expert semantic features as Keys and Values (K, V). In this way, the imaging features can selectively incorporate relevant clinical information according to the observed tumor morphology. This design provides a form of spatially guided regularization, helping the model relate local imaging characteristics, such as internal textural heterogeneity, to clinically meaningful semantic descriptors.

##### 3.1.2.1 Multi-head cross-attention mechanism

Unlike traditional single-head attention, the multi-head mechanism allows the model to simultaneously attend to information from different representation subspaces. In this configuration, the image features *X_1_* as the source of queries and the clinical text features *X*_2_ as the source of keys and values. This configuration allows the network to “query” relevant clinical attributes based on the observed visual morphology. Formally, *X_1_* and *X*_2_ are projected into a shared *d*_model_-dimensional space through *h* sets of learnable linear transformations. For each head i∈{1,…,h}, the attention is computed as:

$Q_{i}=X_{1}W_{i}^{Q},\;K_{i}=X_{2}W_{i}^{K},\;V_{i}=X_{2}W_{i}^{V}$where $W_{i}^{Q}\in R^{dmodel\times d_{k}}$, $W_{i}^{K}\in R^{dmodel\times d_{k}}$, and $W_{i}^{V}\in R^{dmodel\times d_{k}}$ are the weight matrices for Queries, Keys, and Values, respectively, where *d_k_ = d_model_/h:*

$head_{i}=softmax\left(\frac{Q_{i}K_{i}^{T}}{\sqrt{d_{k}}}\right)V_{i}$ The final fused representation *F* is produced by concatenating the outputs of all heads and applying an output transformation $W^{O}\in R^{dmodel\times d\text{model}}$:

##### 3.1.2.2 $F=Concat\left(head_{1},\ldots ,head_{h}\right)W^{O}$Feature aggregation and classification

To condense the spatial-temporal context, we apply a Global Average Pooling (GAP) layer. For a fused volume $F\in R^{d_{model}\times D\times H\times W}$, the global descriptor $z\in R^{d_{model}}$ is calculated as:

$z_{c}\in \frac{1}{D\times H\times W}\sum_{i=1}^{D}\sum_{j=1}^{H}\sum_{k=1}^{W}F_{c,i,j,k}$ Channel index ***c*** facilitates a translation-invariant representation with reduced parameters. Vector ***z*** is fed into the Classification Module (fully connected layers and Softmax) to compute the probabilities for MT and BT.

### 3.2 Experimental setup and model performance indicators

Performance was evaluated via Area Under the Curve (AUC), Matthews Correlation Coefficient (MCC) (16), sensitivity, specificity, accuracy, Positive Predictive Value (PPV), Negative Predictive Value (NPV), F1-score, and Precision-Recall AUC (PR-AUC).To ensure robust internal evaluation and mitigate variance from data splitting, we employed a 10-repeated 5-fold cross-validation strategy. Internal performance metrics were calculated globally based on the aggregated out-of-fold (OOF) predictions, where each patient’s 10 independent validation probabilities were averaged into a single OOF score. The 95% confidence intervals (CIs) for all metrics were generated using 1,000 iterations of patient-level bootstrapping on these OOF predictions. For pairwise model comparisons (e.g., evaluating the added value of multimodal fusion and cross-attention), exact *p*-values were derived directly from the bootstrapped 95% CIs utilizing the method described by Altman and Bland (BMJ, 2011). To strictly address the multiplicity issue arising from multiple comparisons, all derived *p*-values were adjusted using the Benjamini-Hochberg False Discovery Rate (FDR) procedure. Statistical significance was defined as an adjusted *p*-value < 0.05. Decision Curve Analysis (DCA) and an external cohort (*n* = 20) confirmed preliminary clinical utility. Benchmarks included text-only (eXtreme Gradient Boosting(XGboost), Random Forest(RF), Logistic Regression(LR)) and 3D-adapted (ResNet-50, EfficientNet-B4) models, plus a simple concatenation (SC) control. All models were trained from scratch (PyTorch 2.8, Adam, learning rate 1 × 10^-4^, early stopping, RTX 3090 GPU). Robustness to segmentation variance was assessed via nnU-Net Dice stratification. To address the inherent class imbalance within the internal development cohort (250 benign vs. 97 malignant tumors), we employed a class-weighted Cross-Entropy loss function rather than altering the natural data distribution via oversampling or under sampling methods. The class weights were calculated inversely proportional to the class frequencies in the training set to penalize minority class (malignant) misclassifications more heavily. Specifically, the weights were fixed and consistently applied across all evaluated architectures (including the baseline 3D-ResNet, SC model, and our AttenNet) to ensure a rigorously fair and consistent comparison of the architectural improvements. To prevent threshold-optimization bias and ensure rigorous evaluation, diagnostic thresholds were strictly derived without exposing validation or testing data. During the 5-fold cross-validation, the optimal threshold maximizing the Youden index (Sensitivity + Specificity - 1) was calculated exclusively using the training data within each respective fold. This fold-specific threshold was then fixed and applied blindly to the corresponding out-of-fold validation set to compute threshold-dependent metrics, including sensitivity, specificity, F1-score, and accuracy. Furthermore, for the independent external validation cohort, the operating threshold was strictly locked to the optimal Youden index derived from the pooled internal development dataset, ensuring a completely independent and unbiased external evaluation.

For the qualitative interpretability analysis, we employed the Gradient-weighted Class Activation Mapping (Grad-CAM) technique. Rather than introducing a novel attribution formulation, our implementation strictly followed the established Grad-CAM algorithm originally proposed by Selvaraju et al. (17), appropriately adapted for volumetric data as detailed in contemporary 3D medical imaging literature (e.g., Bhavani & Chithra (18)). The gradients of the target class score with respect to the final 3D convolutional feature maps were computed to generate the spatial heatmaps.

## 4 Results

### 4.1 Segmentation performance evaluation

Evaluated across the internal development cohort (*n* = 347), the 3D nnU-Net achieved a median Dice coefficient of 0.827 and a median Hausdorff Distance 95% (HD95) of 5.0 mm. As illustrated by the empirical cumulative distribution function (ECDF) in **Figure 3a**, the majority of internal cases achieved acceptable localization, though approximately 8% of cases presented as low-performing outliers (e.g., Dice < 0.70) due to complex anatomical boundaries. A strong inverse correlation was observed between Dice and HD95 (ρ = −0.752, p = 9.5 × 10^-48^), as shown in **Figure 3b**, further characterizing the relationship between volumetric overlap and boundary distance. Representative examples of segmentation performance are shown in **Figures 3c–f**, illustrating cases with high Dice agreement between the ground-truth and predicted tumor contours. Furthermore, when directly applied to the strictly sequestered external validation cohort (*n* = 20), the frozen nnU-Net maintained robust generalization, yielding a median Dice coefficient of 0.812 (IQR: 0.684–0.872). Given the heavily constrained external sample size, pixel-wise metrics inherently exhibit wider variance; however, this performance confirmed that the upstream module consistently provided adequate and reliable bounding box localization for the downstream classification task.

**Figure 3:**
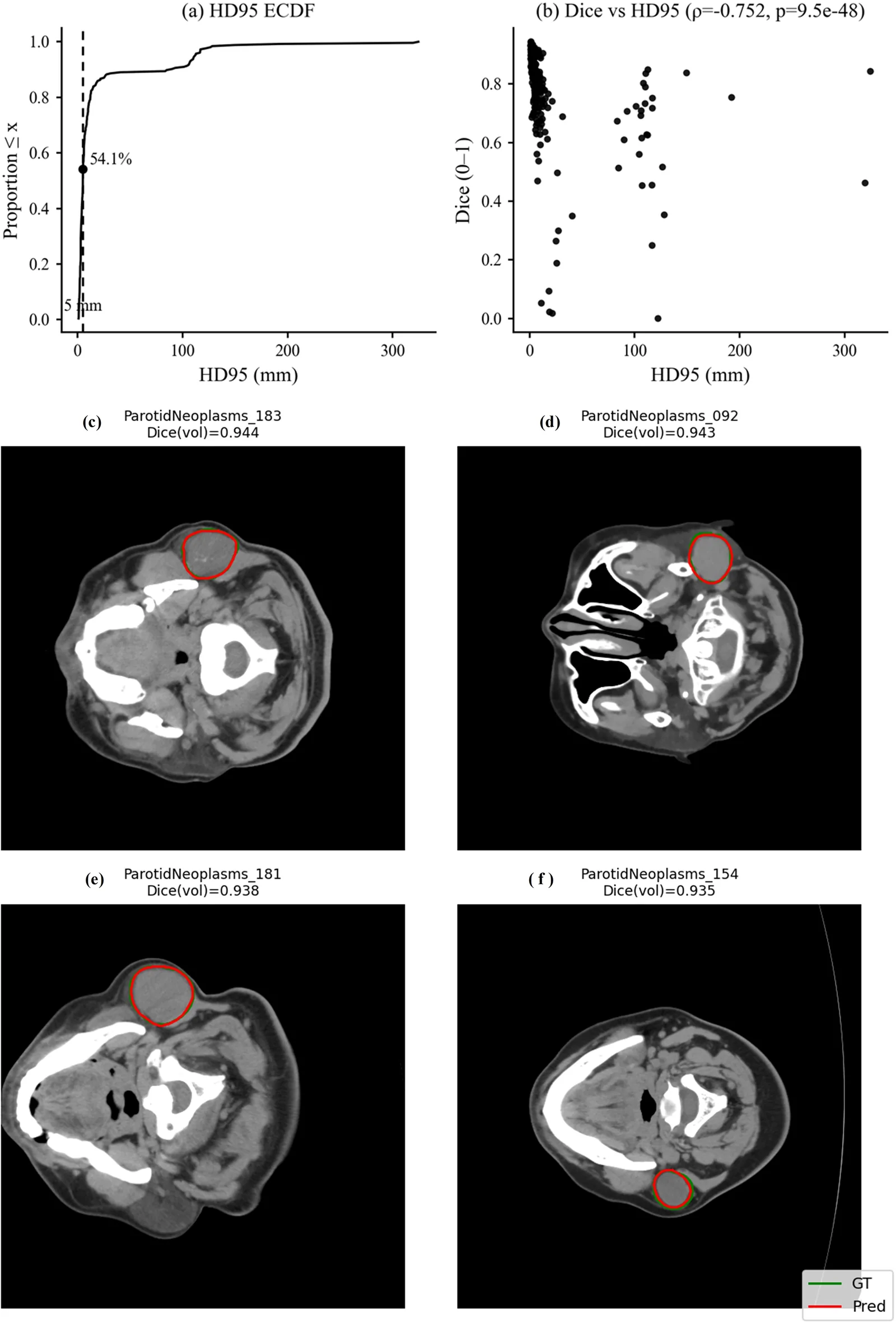
**(a)** Empirical cumulative distribution function (ECDF) of the 95th percentile Hausdorff distance (HD95) for the internal validation cohort. **(b)** Scatter plot showing the correlation between Dice coefficient and HD95 (\(\rho=-0.752\), \(p=9.5\times10^{-48}\)) **(c–f)** Representative axial CT slices from four cases with high volumetric Dice coefficients (0.944, 0.943, 0.938, and 0.935, respectively). Green contours represent ground-truth segmentations, and red contours indicate predictions generated by the model.

### 4.2Performance of expert visual, image-only, and multimodal fusion models

**Table 4** presents the model performance results. The Text_XGB model (using expert features) achieved an AUC of 0.821. 3D-ResNet outperformed 3D-EfficientNet. The AttenNet (based on 3D-ResNet) fusion model achieved the highest AUC (0.908) and MCC (0.677). It also demonstrated the highest sensitivity (0.897), improved specificity (0.832), and superior F1-score (0.770) and PRAUC (0.775).To investigate potential center effects within the development cohort, we stratified the out-of-fold (OOF) predictions by center. The framework maintained robust discriminative performance across institutions, achieving an AUC of 0.912 in Center A (*n* = 314) and 0.881 in Center B (*n* = 33). This consistency indicates that the model generalizes well across the internal centers despite differing scanner hardware (**Table 5**).

**Table 4:** Diagnostic performance of text-only, image-only, and multimodal models.

Model	AUC(95% CI)	ΔAUC vs LR	MCC (95% CI)	ΔMCC vs LR
Text-only model				
Text_XGB	**0.821**(0.771,0.863)	+0.034 (+0.012, +0.057),	**0.529**(0.434,0.618)	+0.005(+0.0000, +0.015),
Text_RF	0.813(0.761,0.857)	+0.026 (+0.006, +0.0470),	0.524(0.428,0.615)	+0.000(+0.000,+0.000),
Text_LR	0.786(0.732,0.832)	-	0.524(0.428,0.615)	-
**Image-only model**		**ΔAUC vs 3D-ResNet**		**ΔMCC vs 3D-ResNet**
3D-ResNet	**0.741**(0.738,0.839)	-	**0.456**(0.366,0.544)	-
3D-EfficientNet	0.664(0.600,0.724)	-0.1268 (-0.2017, -0.0550),	0.269(0.170,0.367)	-0.187(-0.315,-0.060),
**Early fusion model**		**ΔAUC vs 3D-ResNet**		**ΔMCC vs 3D-ResNet**
SC (3D-ResNet)	0.875(0.835,0.912)	+0.0842 (+0.0256, +0.1460),	0.651(0.570,0.729)	+0.195(+0.083,+0.307),
SC (3D-EfficientNe)	**0.904**(0.870,0.934)	+0.1127 (+0.0564, +0.1718),	**0.678**(0.597,0.755)	+0.2218(+0.108,+0.334),
**Cross-attention model**		**ΔAUC vs 3D-ResNet**		**ΔMCC vs 3D-ResNet**
AttenNet (3D-ResNet)	**0.908**(0.873,0.939)	+0.1173 (+0.0593, +0.1762),	**0.677**(0.598,0.750)	+0.221(+0.115,+0.327),
AttenNet (3D-EfficientNe)	**0.890**(0.849,0.926)	+0.0995 (+0.0361, +0.1648),	0.650(0.563,0.734)	+0.194(+0.074,+0.317),

*Performance is reported as AUC and MCC, with corresponding 95% confidence intervals (CI).

The bold values indicate the highest/best values to facilitate easier viewing and comparison.

**Table 5:** Center-stratified diagnostic performance of the fusion framework within the internal development cohort.

Center	AUC(95%CI)	Total cases
A	0.910(0.864,0.926)	314
B	0.880(0.742,0.963)	33

*Data are presented as Area Under the Curve (AUC) with 95% Confidence Intervals (CI). Metrics were calculated using pooled out-of-fold predictions. The wider 95% CI observed for Center B reflects the expected statistical variance due to its limited sample size (*n* = 33).

Statistical comparisons (**Table 6**) revealed that adding expert visual features to the 3D-ResNet baseline via the AttenNet mechanism resulted in a significant performance gain (ΔAUC= +0.1173, adjusted *p* < 0.001) and an improvement in MCC (ΔMCC = +0.2213, adjusted *p* < 0.001). The transition from 3D-EfficientNet to the early fusion SC (3D-EfficientNet) also showed a substantial improvement (ΔAUC = +0.2395, adjusted *p* < 0.001). Although AttenNet (3D-ResNet) marginally outperformed the SC (3D-ResNet) model in absolute terms (ΔAUC = +0.0331), the difference was not statistically significant (adjusted *p* = 0.0830).

**Table 6:** Pairwise comparison of performance across model architectures.

Comparison	ΔAUC(95%CI)	Adjusted p-value (AUC)	ΔMCC(95%CI)	Adjustedp-value (MCC)
**Text_XGB** vs Text_LR	**+0.0342 (+0.0120, +0.0572)**	**0.0066**	+0.0048(+0.0000,+0.014)	0.3247
**Text_RF** vs Text_LR	**+0.0262 (+0.0063, +0.0468)**	**0.0176**	+0.0000(+0.0000,+0.000)	1.0000
Text_XGB vs Text_RF	+0.0071 (-0.0009, 0.0146)	0.0828	+0.0048 (0.0000, 0.0149)	0.3247
3D-EfficientNet vs **3D-ResNet**	**-0.1268 (-0.2017, -0.0550)**	**0.0019**	**-0.1865(-0.3145,-0.0603)**	**0.0089**
**SC(3D-EfficientNe)** vs 3D-ResNet	**+0.1127 (+0.0564, +0.1718)**	**< 0.001**	**+0.2218(+0.1084,+0.333)**	**< 0.001**
**SC(3D-ResNet)** vs 3D-ResNet	**+0.0842 (+0.0256, +0.1460)**	**0.0112**	**+0.1950(+0.0834,+0.307)**	**0.0017**
**AttenNet (3D-ResNet)** vs 3D-ResNet	**+0.1173 (+0.0593, +0.1762)**	**< 0.001**	**+0.2213(+0.1154,+0.327)**	**< 0.001**
AttenNet(3D-ResNet) vs SC(3D-ResNet)	+0.0331 (-0.0021, +0.0677)	0.0828	+0.0263(-0.0283,+0.0820)	0.4277
**SC(3D-EfficientNet)** vs 3D-EfficientNet	**+0.2395 (+0.1750, +0.3075)**	**< 0.001**	**+0.4083(+0.2833,+0.535)**	**< 0.001**
AttenNet(3D-EfficientNet) vs SC(3D-EfficientNet)	-0.0132 (-0.0609, +0.0338)	0.5848	-0.0278 (-0.1495,+0.0873)	0.7099
AttenNet(3D-ResNet) vs AttenNet(3D-EfficientNet)	+0.0172 (-0.0011, +0.0368)	0.0828	+0.0263(-0.0195,+0.0797)	0.4107

*Pairwise comparisons between model architectures are presented using differences in AUC (ΔAUC) and MCC (ΔMCC), with corresponding 95% confidence intervals (CI). Exact *p*-values were derived from the bootstrapped 95% CIs utilizing the Altman-Bland method. To address multiplicity, all *p*-values were adjusted using the Benjamini-Hochberg False Discovery Rate (FDR) procedure. Bold values indicate statistical significance (*p* < 0.05).

Robustness to segmentation errors was evaluated by stratifying the cohort based on nnU-Net Dice coefficients. AttenNet and SC remained stable across all groups, while image-only models exhibited a decline in performance in the low-Dice group, whereas fusion models exhibited greater robustness (**Figure 4**).

**Figure 4:**
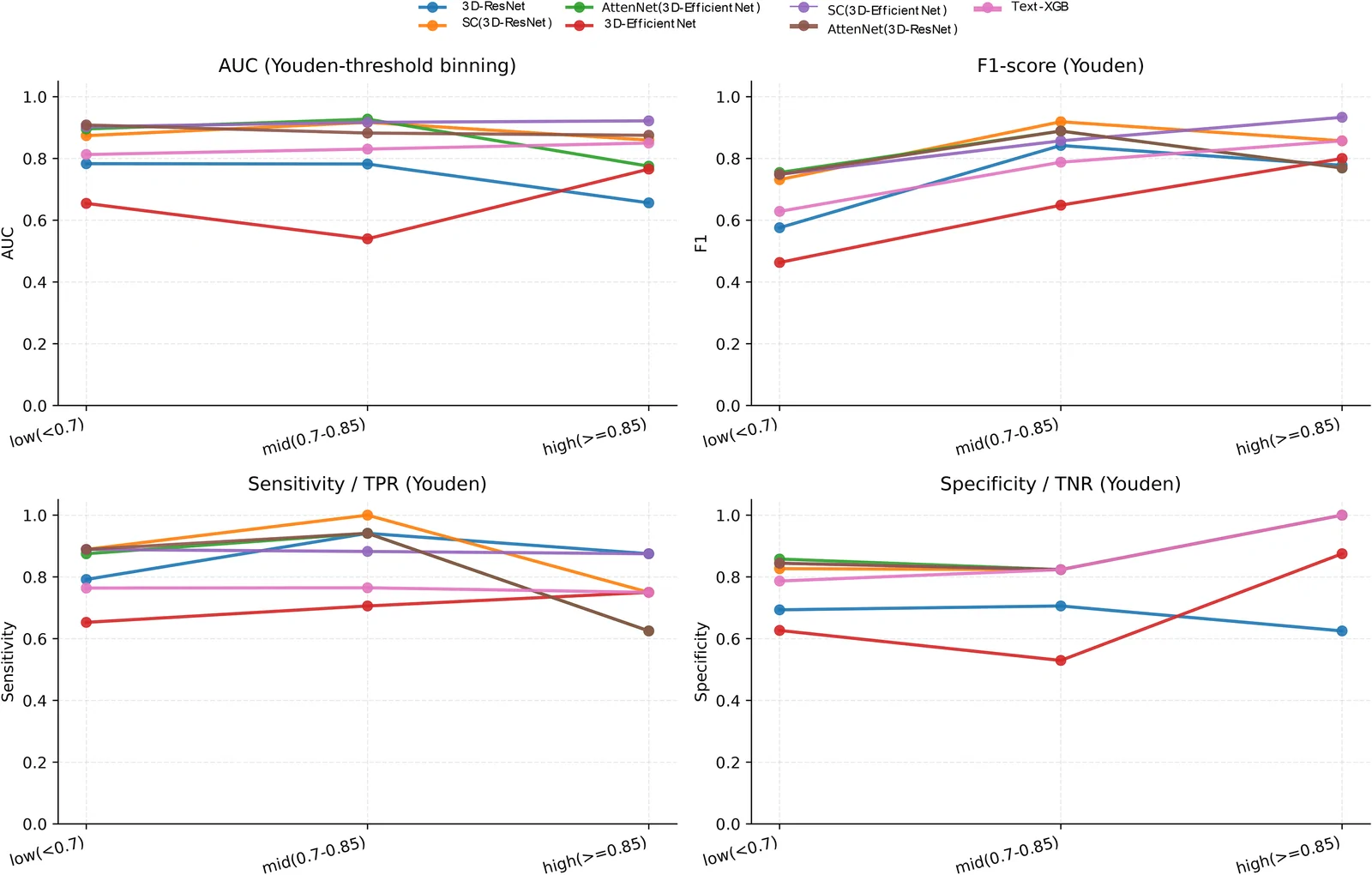
Classification performance across different segmentation quality levels. The models are evaluated based on Dice coefficient groups: low (<0.7), mid (0.7-0.85), and high (≥0.85). Metrics include AUC, F1-score, Sensitivity, and Specificity. The results demonstrate the robustness of SC and AttenNet variants across varying segmentation accuracies.

Nine models were evaluated via ROC analysis. AttenNet (3D-ResNet) had the highest AUC (0.908), followed by SC (3D-EfficientNet) (0.904) and AttenNet (3D-EfficientNet) (0.890). Traditional models (XGBoost, RF, LR) showed moderate AUCs (0.786–0.821), while 3D-EfficientNet yielded the lowest value (0.664). DCA indicated that AttenNet (3D-ResNet50) had the best net benefit across the thresholds 0.2–0.6.While other models like SC(3D-EfficientNet) and AttenNet(3D-EfficientNet) showed clinical value, their net benefits were more volatile or declined sharply at higher thresholds (**Figure 5**).

**Figure 5:**
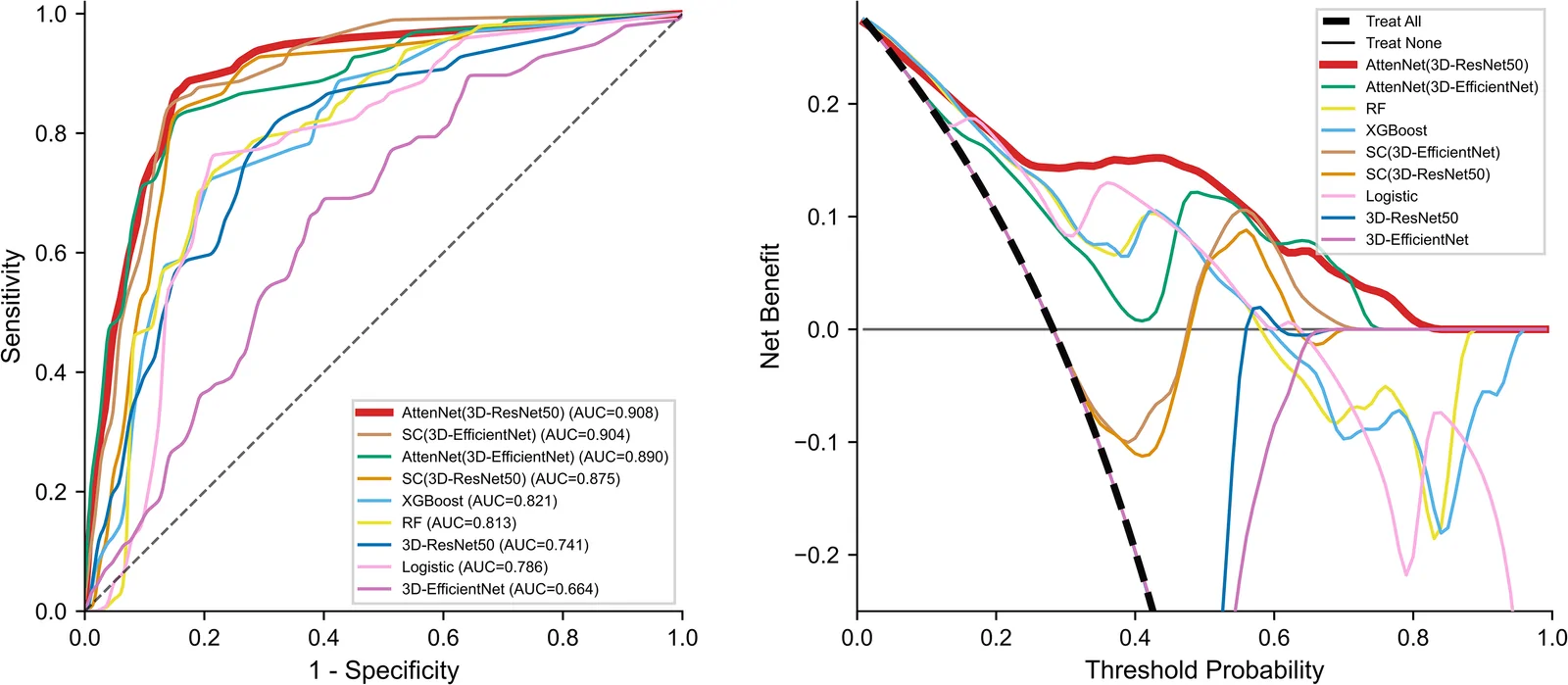
Evaluation of nine predictive models. (Left) ROC curves show AttenNet (3D-ResNet50) achieved the highest discriminative power (AUC = 0.907). (Right) DCA demonstrates its superior clinical utility over alternatives.

### 4.3 Ablation study

As summarized in **Table 7**, both modified architectures, SC(3D-ResNet) and AttenNet(3D-ResNet), showed improved predictive performance compared with the baseline models. The SC(3D-ResNet) achieved an AUC of 0.875, which was higher than those of standard 3D-ResNet (AUC = 0.741) and the text-based Text_XGB model (AUC = 0.821). The proposed AttenNet(3D-ResNet) achieved the highest AUC of 0.908 among the evaluated models, with the difference reaching statistical significance after adjustment (adjusted p < 0.05).The analysis of model complexity, including the number of parameters (Params) and computational cost (FLOPs), provides further information regarding the computational characteristics of the proposed architectures. SC(3D-ResNet) achieved an absolute AUC increase of more than 13% compared with standard 3D-ResNet, while introducing minimal changes in computational requirements. Specifically, FLOPs increased only slightly from 6.082 G to 6.083 G, and the number of parameters increased from 60.731 M to 61.005 M. AttenNet(3D-ResNet), which achieved the highest AUC, contained 70.451 M parameters and required 6.313 G FLOPs, corresponding to an approximately 3.8% increase in FLOPs relative to the baseline model. Overall, these findings suggest that the proposed architectures can improve predictive performance while maintaining a relatively moderate computational cost, indicating a potentially favorable balance between model performance and computational requirements.

**Table 7:** Performance comparison and computational complexity of different models.

Models	AUC(95%CI)	Adjusted *p*-value	Params (M)	FLOPs (G)
Text_XGB	0.821(0.771,0.863)			
3D-ResNet	0.741(0.738,0.839)		60.731	6.082
SC(3D-ResNet)	0.875(0.835,0.912)		61.005	6.083
AttenNet(3DResNet)	**0.908**(0.873,0.939)	**<0.05**	70.451	6.313

*AUC: Area Under the Receiver Operating Characteristic Curve; CI: Confidence Interval.Params (M): Number of trainable parameters in millions, indicating the model complexity/size. FLOPs (G): Floating-point operations in billions, indicating the computational cost. The Params and FLOPs for Text_XGB are not listed, as it is a tree-based machine learning model rather than a deep neural network, making these specific complexity metrics incomparable. *Adjusted p-value* indicates the statistical significance of the difference between AttenNet(3DResNet) and the baseline model (e.g., Text_XGB or 3D-ResNet), with *p* < 0.05 considered statistically significant.

The bold values indicate the highest/best values to facilitate easier viewing and comparison.

To examine the relationship between the different modalities, we compared the prediction consistency of the image-only (3D-ResNet), semantic-only (Text-XGB), and fusion (AttenNet) models using Pearson correlation coefficients (**Figure 6**). The predictions from the image-only and semantic-only models showed a moderate correlation (Pearson *r* = 0.56), suggesting that the two modalities provide partly different information for prediction. In comparison, the predictions of the fusion model were more closely correlated with those of the semantic-only model (Pearson *r* = 0.95), while also showing a moderate-to-strong correlation with the image-only model (Pearson *r* = 0.79). This pattern suggests that the fusion model retains a substantial contribution from the semantic features while also incorporating information from the imaging features. Thus, the prediction behavior of AttenNet appears to reflect contributions from both modalities rather than being entirely driven by the semantic features alone.

**Figure 6:**
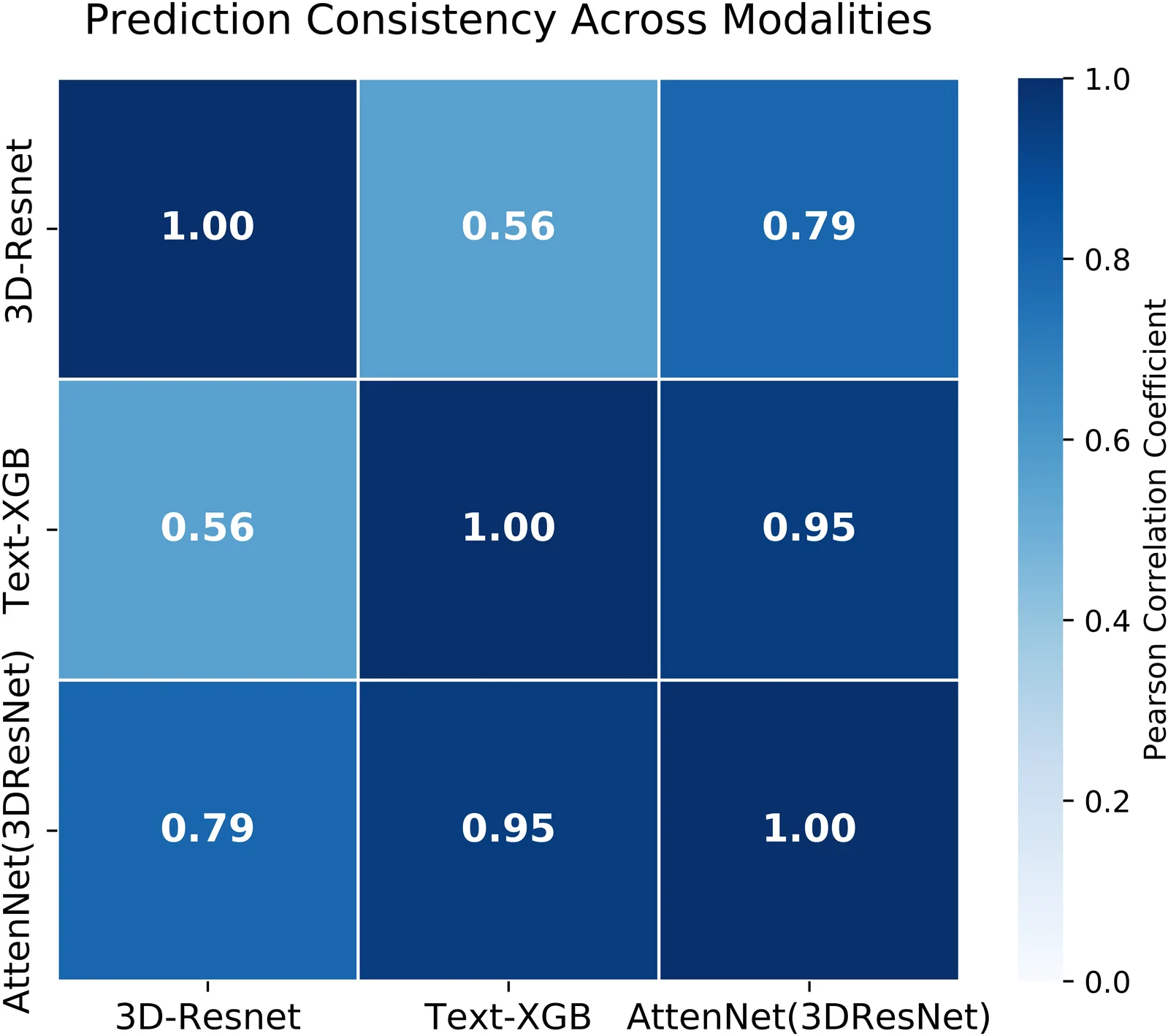
Prediction consistency and modality correlation across the evaluated diagnostic models. Pearson correlation heatmap of out-of-fold predictions from 3D-ResNet, Text-XGB, and AttenNet. AttenNet showed stronger correlations with both Text-XGB (r = 0.95) and 3D-ResNet (r = 0.79), whereas the correlation between the two single-modality models was moderate (r = 0.56).

### 4.3 Grad-CAM visualization of different models

Grad-CAM visualizations across five cases (**Figure 7**) suggest variations in how the models focus. AtteNet(3D-ResNet) appears to generally align its activation with relevant tumor features, such as boundaries and internal regions. In contrast, the 3D-ResNet and SC(3D-ResNet) models tend to exhibit somewhat more dispersed or fragmented activation patterns. For instance, they occasionally focus on peripheral areas, the mandible, or extend into the background. Overall, these observations suggest that AtteNet may provide a relatively more focused and consistent localization of the lesions.

**Figure 7:**
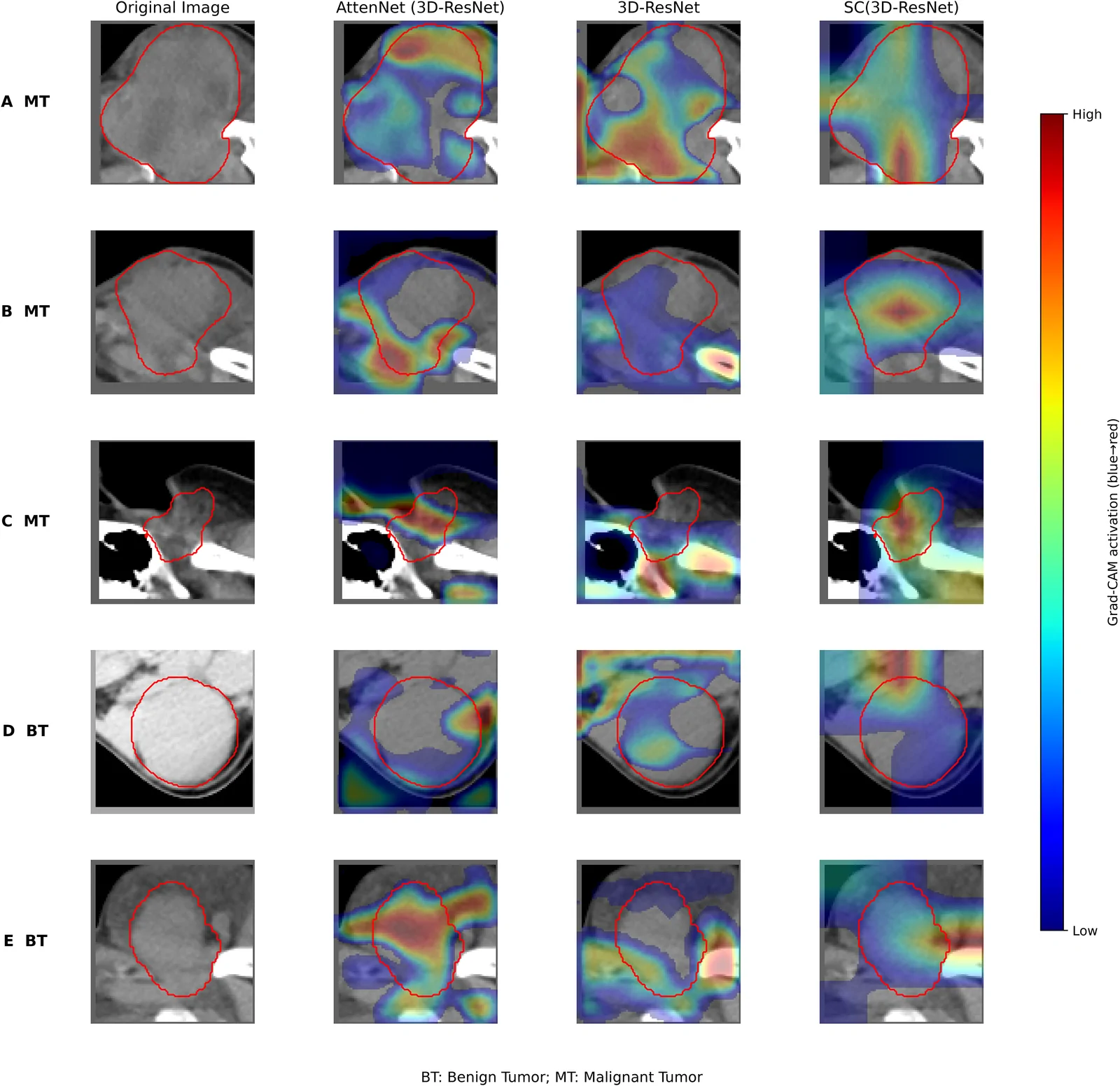
Grad-CAM comparison of 3D-ResNet models. First column: original images with ROI. Rows A–C: malignant tumors (MT); D–E: benign (BT). Heatmaps show attention maps of AtteNet, 3D-ResNet, and SC(3D-ResNet). Color scale (blue to red) indicates activation intensity.

### 4.4 External validation

In a preliminary external validation cohort (*n* = 20), the fusion models SC (3D-ResNet50) and AttenNet achieved AUCs of 0.910 and 0.820, respectively, suggesting a potential advantage over the baseline 3D-ResNet50 (AUC: 0.560) (**Table 8**; **Figure 8B**). The exact patient counts for true positive, true negative, false positive, and false negative predictions in the external cohort are detailed in the confusion matrices (**Figure 8A**). The corresponding ROC curves are shown in **Figure 8B**, and the decision curve analysis is presented in **Figure 8C** these results exhibit wide confidence intervals due to the limited sample size. Therefore, these findings should be interpreted cautiously as a proof-of-concept for the multimodal approach, rather than definitive confirmation of clinical viability.

**Table 8:** Comparison of diagnostic performance metrics among deep learning models, machine learning, and clinical experts.

Model / Radiologist	AUC(95%CI)	F1score(95%CI)	MCC(95%CI)	Sensitivity(95%CI)	Specificity(95%CI)
3D-ResNet50	0.565(0.283,0.828)	0.456(0.143,0.727)	0.108(-0.373,0.522)	0.400(0.100, 0.714)	0.704 (0.400, 1.000)
AttenNet (3D-ResNet50)	0.820(0.600,0.980)	0.793(0.533, 0.957)	0.604 (0.204, 0.905)	0.803 (0.500, 1.000)	0.802 (0.539, 1.000)
SC (3D-ResNet50)	**0.910**(0.747,1.000)	0.789 (0.545, 0.960)	0.597 (0.206, 0.905)	0.796 (0.500, 1.000)	0.802 (0.539, 1.000)
XGBoost	0.560 (0.298, 0.845)	0.574 (0.300, 0.800)	-0.001(-0.459,0.464)	0.703 (0.385, 1.000)	0.296(0.000, 0.6000)
Radiologist A	–	0.723 (0.444, 0.917)	0.500(0.0890, 0.817)	0.697 (0.375, 1.000)	0.802 (0.539, 1.000)
Radiologist B	–	0.767 (0.500, 0.948)	0.609 (0.218, 0.905)	0.702 (0.375, 1.000)	0.898 (0.667, 1.000)

*Data are presented as the mean value followed by the 95% confidence interval (CI) in parentheses. Experts A and B represent independent assessments by two senior clinicians with 15 years of experience.

The bold values indicate the best performance among the evaluated models for each metric.

**Figure 8:**
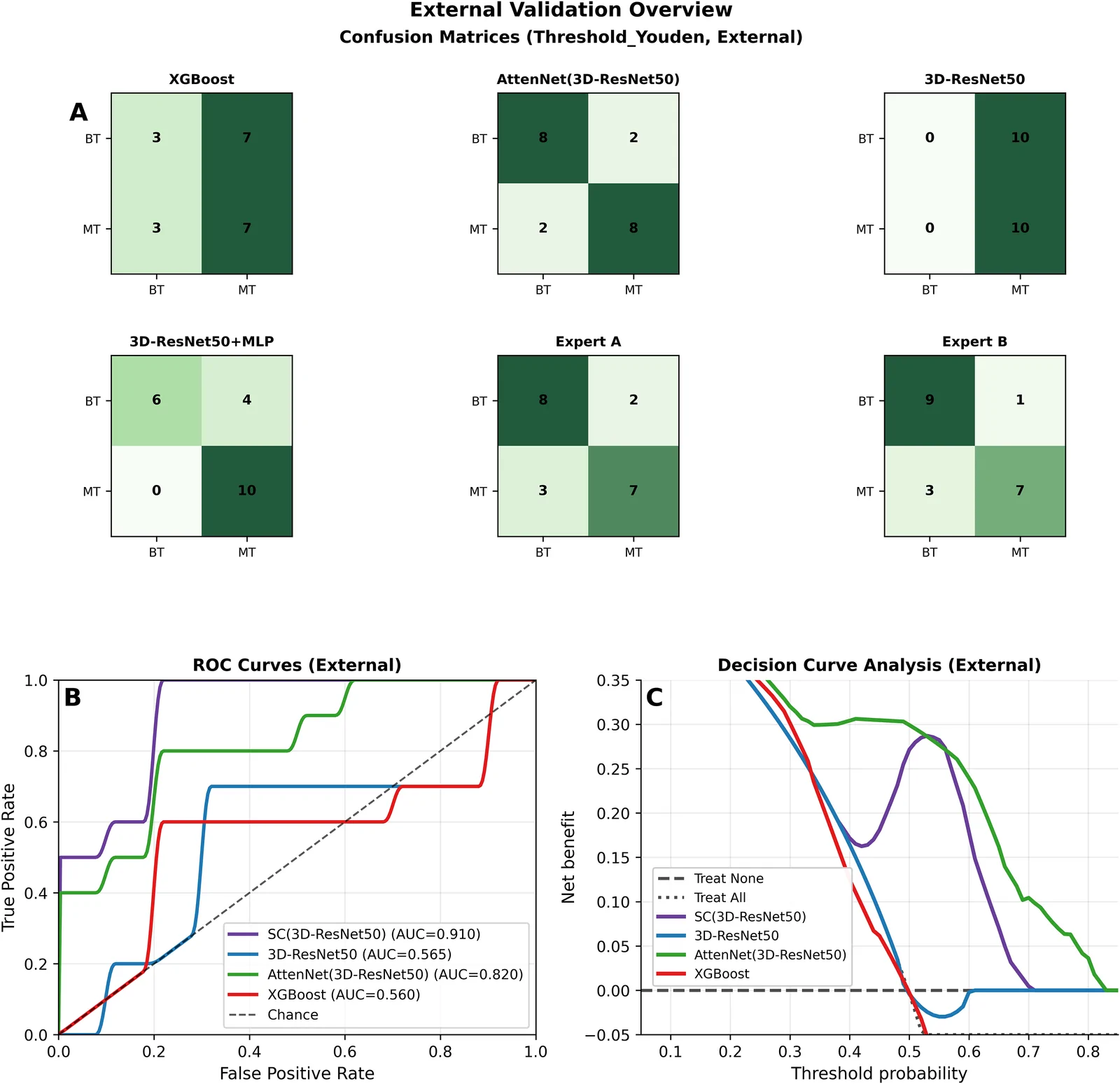
Performance of models and experts in external validation. **(A)** Confusion matrices for four computational models (thresholds optimized via Youden Index) and two experts (BT: benign; MT: malignant). **(B)** ROC curves comparing models’ diagnostic efficacy. **(C)** DCA evaluating clinical net benefit across threshold probabilities.

## 5 Discussion

This performance provided the coarse lesion localization sufficient for ROI extraction and classification. In a prospective real-world clinical workflow, we envision a clinician-in-the-loop paradigm: the nnU-Net module first automatically delineates the ROI, after which the attending radiologist briefly evaluates the non-contrast CT to input the four categorical features (density, shape, margin, and central necrosis) via a user interface. AttenNet subsequently processes these modalities to output a malignancy probability, serving as a rapid second reader.

To visually illustrate the potential robustness of the framework to spatial localization errors in the upstream segmentation network, **Figure 9** presents representative cases with low segmentation performance (Dice < 0.6). In cases of severe oversegmentation (**Figures 9a, b**) and undersegmentation (**Figures 9c, d**), image-only models may be more susceptible to localization errors. In contrast, AttenNet maintained relatively accurate diagnostic predictions in these examples, potentially benefiting from the complementary information provided by the expert semantic features, which may serve as protective priors when segmentation quality is limited.

**Figure 9:**
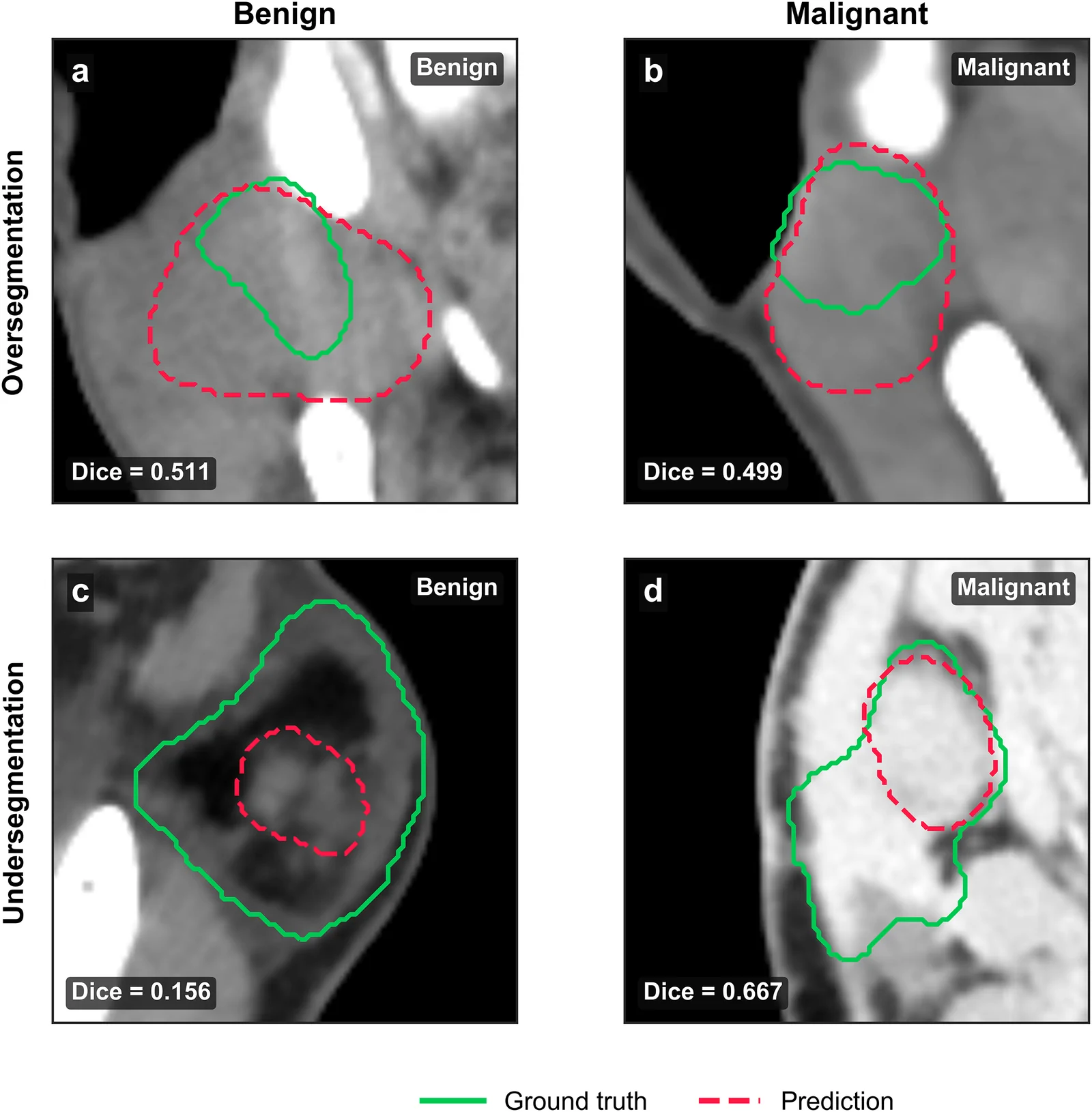
Representative segmentation cases across Dice levels. Representative cases of oversegmentation **(a–b)** and undersegmentation **(c–d)**, showing the ground-truth contours (green) and predicted contours (red dashed). Dice scores are indicated in each panel.

Multimodal fusion showed potentially useful performance compared with single-modal models (e.g., XGBoost and 3D-ResNet) in the internal evaluation. The expert-guided AttenNet achieved an AUC of 0.908, with generally consistent performance across the evaluated metrics. These findings provide preliminary evidence of the model’s potential for supporting auxiliary diagnosis. However, the results were obtained from internal testing, and formal reader-assistance studies are needed to determine whether such performance could translate into meaningful clinical benefits. Calibration analysis was also conducted to assess probabilistic reliability in the internal evaluation. As shown in **Figure 10**, the uncalibrated pure-image baseline models (e.g., 3D-ResNet50, Brier score = 0.274) showed relatively high prediction confidence despite imperfect calibration. AttenNet achieved a lower Brier score (0.183), indicating better agreement between predicted probabilities and observed outcomes in this internal evaluation. This observation suggests that multimodal integration may be associated with differences in probabilistic calibration. However, the observed difference should be interpreted cautiously, as it does not establish a direct contribution of the cross-attention mechanism to calibration performance. Our paired comparisons showed that the quantitative diagnostic gain of MHAF over simple concatenation (SC) was relatively marginal. The motivation for exploring cross-attention therefore extends beyond demonstrating statistical superiority. As theoretically formulated, the MHAF framework may help incorporate semantic information into the attention mechanism and facilitate the integration of complementary imaging features. It may also help reduce the risk of over-reliance on semantic information. Nevertheless, these potential benefits remain hypotheses, and the current internal evaluation does not provide sufficient evidence to establish that cross-attention offers more robust or geometrically grounded multimodal integration than simpler fusion strategies. Overall, these internal findings suggest that AttenNet may provide complementary information for diagnostic prediction and probabilistic estimation. However, the results should be considered exploratory, and their generalizability to independent populations and clinical practice remains uncertain. Further validation in larger, independent cohorts, including external calibration assessment and formal reader-assistance studies, will be necessary to assess the reproducibility and potential clinical relevance of these observations.

**Figure 10:**
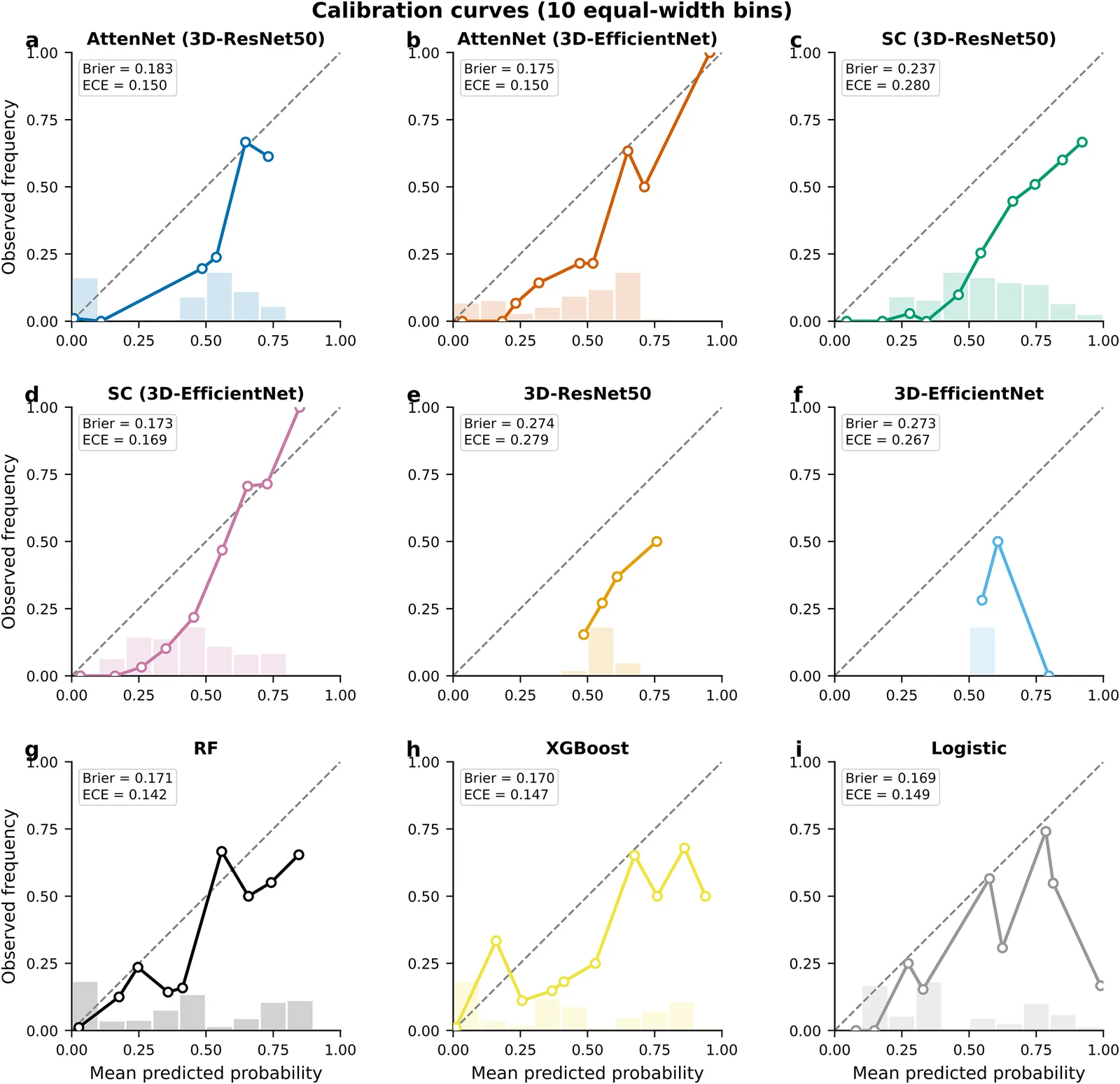
Calibration curves of the evaluated models in the internal cohort. Calibration curves for the evaluated models in the internal cohort, using 10 equal-width probability bins. The dashed diagonal line represents perfect calibration. Brier scores and expected calibration errors (ECE) are shown for each model. Panels **(a–f)** show deep learning models, including AttenNet and simple-concatenation (SC) fusion models, while panels **(g–i)** show machine-learning models based on structured semantic descriptors.

To explore the respective contributions of the multi-head attention mechanism and the two modalities, a *post-hoc* feature knockout ablation analysis was performed (**Supplementary Table S7.docx**). During inference, masking either the cross-attention spatial output or the expert semantic features was associated with a marked reduction in discriminative performance, with AUC decreasing from 0.908 to approximately 0.53 and 0.52, respectively. These exploratory findings suggest that both imaging and expert semantic features may contribute to the model’s predictions, and that their interaction may be relevant to the observed diagnostic performance. However, the results should be interpreted cautiously, as *post-hoc* feature masking may disrupt the learned feature representations and does not necessarily reflect the independent contribution of each modality during normal inference. Therefore, this analysis provides preliminary evidence of multimodal feature dependence but does not establish genuine synergistic fusion or rule out all forms of modality reliance. Further validation using complementary ablation strategies and independent cohorts is warranted.

Notably, subgroup analysis based on segmentation quality underscores the potential synergy between semantic and spatial features. Results indicate that while 3D deep learning exhibits reduced performance when segmentation accuracy declines, fusion models demonstrate robustness against such degradation by utilizing “expert knowledge” as a regularization mechanism. To address potential confounders in our Dice-stratified subgroup analysis, we statistically evaluated tumor size and class distribution across the three strata (High: *n* = 200; Medium: *n* = 120; Low: *n* = 27). Chi-square testing confirmed no significant difference in the benign-to-malignant ratio across groups (*p* = 0.1647), ruling out class imbalance as a confounder. However, Kruskal-Wallis testing revealed that the Low-Dice group comprised significantly smaller tumors by volume (median 9.47cm^3^ vs. 17.82cm^3^ in the High-Dice group, *p* < 0.001. This aligns with the mathematical nature of the Dice metric, which is intrinsically highly sensitive to boundary errors in small targets. Rather than invalidating the analysis, this biological reality highlights the exact clinical scenario where our fusion model excels: for small, hard-to-segment lesions where spatial image features are compromised, the integration of expert semantic features provides critical diagnostic regularization, preventing the severe performance degradation observed in image-only models. This mechanism preserves diagnostic stability despite segmentation fluctuations, indicating significant clinical utility in complex clinical settings.

In the rapidly evolving realm of medical imaging, integrating Multimodal Explainable Artificial Intelligence (XAI) has become indispensable for bridging the chasm between intricate algorithmic predictions and clinical decision-making (19–21). While the primary objective of this study is binary classification rather than image segmentation, interpreting exactly how the model formulates its diagnostic predictions is critical for clinical trust. Multi-modal fusion appears to offer advantages over single-modal approaches (e.g., XGBoost, 3D-ResNet) when addressing complex cases. Although both Simple Concatenation (SC) and AttenNet demonstrated highly significant performance improvements over image-only baselines (adjusted p < 0.001), the quantitative difference between the two fusion strategies was not statistically significant. However, a primary contribution of the attention mechanisms lies in enhancing qualitative interpretability. Exploratory 3D Grad-CAM visualizations suggested differences in the spatial focus of the evaluated models. In the five representative cases examined, AttenNet (3D-ResNet) showed attention in regions corresponding to tumor boundaries and internal heterogeneous areas in several cases. These regions may be relevant to the imaging assessment of parotid tumors, as irregular margins and internal heterogeneity can be observed in malignant lesions, whereas benign tumors may more often present with well-circumscribed margins. However, these visual observations do not establish that the model learned specific pathological features or that the highlighted regions directly contributed to accurate predictions. Moreover, the examples were selected for illustration and do not represent a systematic assessment of all cases, including incorrect predictions. Therefore, the Grad-CAM findings should be considered exploratory and hypothesis-generating. Further studies incorporating quantitative localization metrics, radiologist agreement, and occlusion- or insertion/deletion-based analyses are needed to assess the reliability, stability, and clinical relevance of the observed attention patterns.

While external validation (*n* = 20) yielded F1 and MCC scores comparable to participating radiologists, this is best viewed as a preliminary proof-of-concept. It is important to note that in this limited external cohort, the SC model nominally outperformed AttenNet (AUC 0.910 vs. 0.820). Given the extremely small sample size (*n* = 20), a shift in prediction for merely one or two cases can cause drastic AUC fluctuations. Therefore, this difference is highly sensitive to sample variance and does not definitively establish the generalizability superiority of either fusion method. We acknowledge that the quantitative improvements of the proposed MHAF mechanism over simple concatenation (SC) were relatively limited and did not reach statistical significance, as the 95% CI encompassed zero. In addition, SC achieved a numerically higher AUC in the external validation set (*n* = 20), indicating that simpler fusion architectures can also achieve competitive discriminative performance. Therefore, we do not claim that cross-attention provides absolute quantitative superiority over SC in this dataset. Instead, we explored whether the MHAF mechanism might offer complementary benefits in feature integration and model interpretability. In the current internal evaluation, Grad-CAM visualizations showed differences in the spatial focus of the evaluated models. The SC model sometimes showed attention in non-specific background regions, whereas AttenNet (3D-ResNet) showed attention in regions corresponding to tumor boundaries and internal heterogeneous areas in several representative cases. These observations may indicate differences in the way the models use imaging and semantic information. However, they do not establish that SC relies on statistical shortcuts or that MHAF acts as a clinical regularizer. From a methodological perspective, incorporating expert semantic features into the attention mechanism may provide a way to explore the interaction between clinical prior information and imaging features. In principle, this design could help the model consider anatomically relevant information during feature integration. Nevertheless, whether MHAF improves the geometric grounding, interpretability, or robustness of multimodal predictions remains uncertain. The current Grad-CAM examples are exploratory and do not establish that the highlighted regions correspond to true pathological indicators or that the model’s predictions are based on clinically valid reasoning. Thus, the potential value of the proposed attention mechanism in this study lies in its exploratory investigation of multimodal feature integration, rather than in demonstrating definitive quantitative superiority over simpler fusion strategies. Further validation using systematic explanation assessment, quantitative localization analyses, and larger independent cohorts is needed to determine whether these potential benefits can be reproduced and whether they translate into clinically meaningful improvements. External calibration was also evaluated (**Figure 11**). However, the external cohort was small (*n* = 20), with approximately four cases per bin, which limited the reliability of the calibration estimates. These results should therefore be interpreted with caution. Larger independent cohorts are needed to further evaluate model calibration and its generalizability across different patient populations. Additionally, Decision Curve Analysis (DCA) indicates that AttenNet maintains a relatively stable and superior net benefit across varying thresholds. Of note, we focused on the threshold probability range of 0.2 to 0.6 when interpreting the DCA which reflects the realistic clinical risk-tolerance boundaries for parotid tumor management. In this clinical scenario, extreme thresholds are impractical: extremely low thresholds may increase the risk of overtreatment (e.g., unnecessary facial nerve sacrifice for benign lesions), while extremely high thresholds may increase the risk of undertreatment of malignancies. Within this clinically meaningful threshold range (0.2–0.6), the AttenNet framework showed a higher net clinical benefit than baseline models and the “treat-all/treat-none” strategies. These findings suggest its potential value in supporting surgical planning, although further clinical validation is needed. This cross-dataset consistency may mitigate the limitations of the small sample size, highlighting the potential of attention mechanisms in capturing generalizable features.

**Figure 11:**
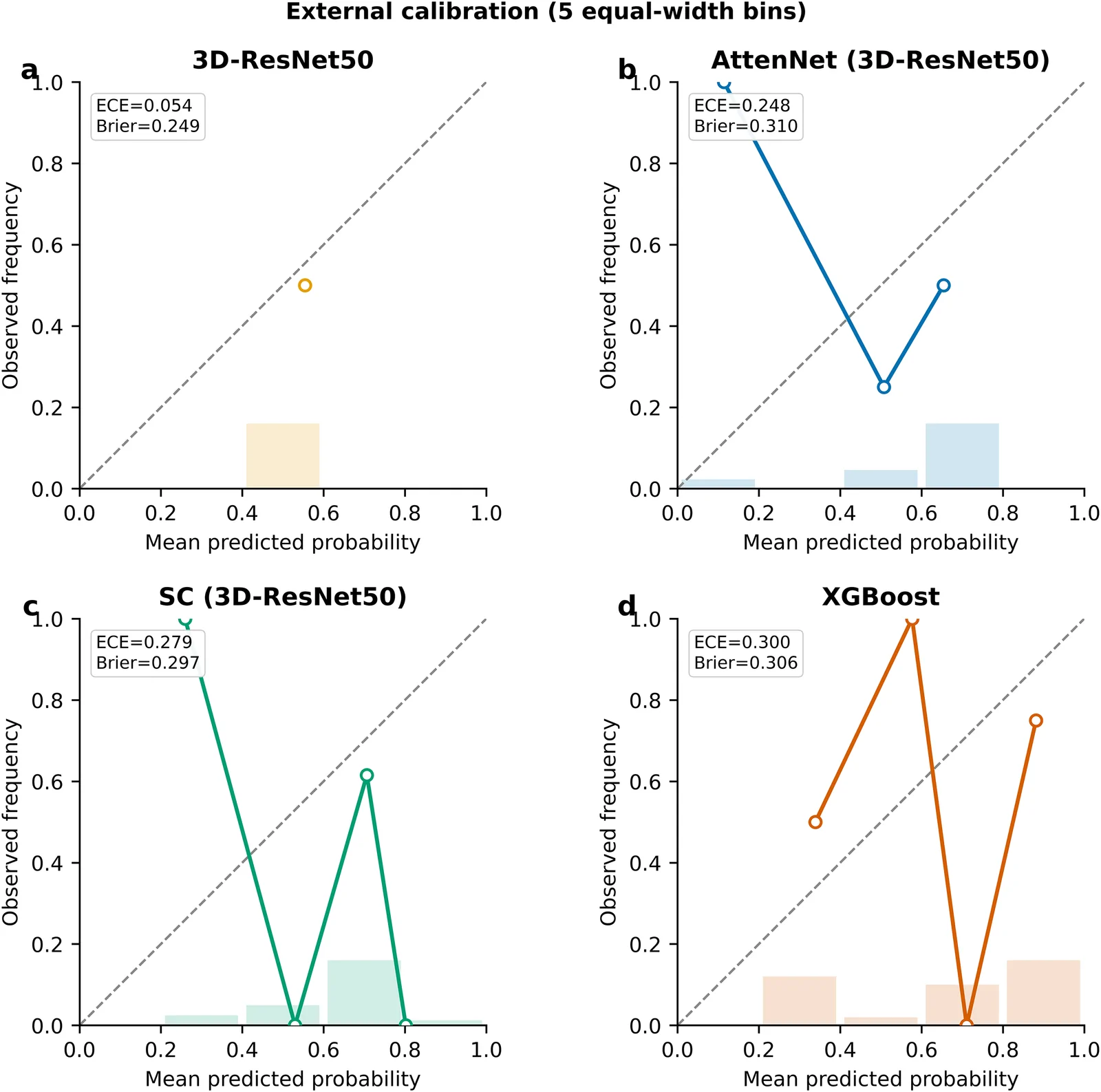
External calibration curves. External calibration curves for the evaluated models using 5 equal-width probability bins. The dashed diagonal line represents perfect calibration. Brier scores and expected calibration errors (ECE) are shown for each model. Panels **(a–d)** show the 3D-ResNet50, AttenNet (3D-ResNet50), SC (3D-ResNet50), and XGBoost models, respectively.

Although the current framework relies on consensus-derived features, our analysis revealed substantial to almost perfect inter-rater agreement among the three experts (Fleiss’ Kappa ranging from 0.74 to 0.85; **Supplementary Table S1**). This high level of concordance suggests that the semantic features input into AttenNet remain relatively stable across individual readers, theoretically minimizing the risk of significant performance degradation when applied by a single radiologist in routine practice.

However, the small external validation sample (*n* = 20) results in wide confidence intervals and potential random fluctuations. While the multimodal strategy shows promising clinical gains, its generalizability and long-term robustness require further validation in larger, multicenter datasets.

Unlike previous studies depending on contrast-enhanced MRI and CT (8, 9), our method uses plain CT alone. While Hu et al. (10) utilized manual 2D non-contrast CT analysis, we employ nnU-Net (15) for automated 3D volumetric segmentation. Although bounding box enlargement captures peritumoral infiltration, it risks introducing background anatomical biases. Despite demonstrated model robustness, future work must disentangle peritumoral contextual effects from true lesional features.

This study has several limitations. First, a notable limitation pertains exclusively to the internal evaluation due to the upstream nnU-Net training strategy. Because the segmentation module was trained using the entire internal development cohort (347 patients from Centers A and B) rather than employing a strict out-of-fold segmentation framework, it likely generated overly optimized Regions of Interest (ROIs) for these specific cases. Consequently, the downstream classification metrics obtained during internal cross-validation may represent optimistic upper bounds, as the classifier benefited from artificially precise spatial localization. However, it is crucial to emphasize that this limitation is strictly confined to the internal cohort. The external validation cohort (Center C) was completely sequestered from the nnU-Net training phase, ensuring that the external performance metrics authentically reflect the model’s robustness against unrefined, out-of-distribution spatial inputs in a real-world setting. Therefore, the external performance metrics remain a valid proof-of-concept for the fusion framework’s real-world robustness against unrefined, out-of-distribution upstream segmentation errors. Specifically, nnU-Net was utilized strictly for coarse spatial localization (bounding box cropping). Because the classification module (AttenNet) was trained independently within each cross-validation fold—and received only the raw CT intensities of the expanded bounding box rather than the explicit segmentation boundaries—it was forced to independently extract lesional and peritumoral features. Therefore, the definitive diagnostic probability relies entirely on the cross-validated classifier’s internal feature representations, preserving the statistical integrity of our internal evaluations. Future pipelines should ensure strict out-of-fold isolation for both segmentation and classification stages. Second, the modest sample size (total *n* = 367, external *n* = 20) and the rarity of certain histological subtypes (detailed in **Supplementary Table S5**) precluded specific subgroup analyses and may restrict generalizability (22, 23). Clinical interpretation of our external validation metrics requires caution. The external cohort was artificially balanced to a 50% malignancy prevalence (10 benign vs. 10 malignant) to stress-test the model’s diagnostic capacity on a limited sample. Consequently, prevalence-dependent metrics such as Accuracy and Positive Predictive Value (PPV) may not directly translate to real-world clinical environments where malignant parotid tumors are significantly less common. However, prevalence-independent metrics, notably the ROC-AUC, PR-AUC, Sensitivity, and Specificity, reliably confirm the model’s robust discriminative capabilities across diverse cohorts. Third, while class imbalance was mitigated using stratified cross-validation and weighted loss, the reliance on manually assessed semantic features introduces inherent subjectivity and potential inter-observer variability (24–26). Although strict blinding reduced the risk of surrogate labeling, generalizability across radiologists with varying experience levels remains a concern. Forth, the model’s performance was evaluated using consensus semantic features rather than individual assessments. While high inter-observer agreement was confirmed, relying on consensus may not perfectly emulate a typical clinical workflow involving a single reader. Future prospective reader-assistance studies must assess the framework’s diagnostic robustness using individual radiologist inputs. Furthermore, while the detailed histological subtype distribution for the internal development cohort is rigorously documented (**Supplementary Table S5**), the precise pathological subtyping for the external validation cohort (*n* = 20) could not be fully retrieved due to the constraints of retrospective multi-center data sharing, where only binary (benign/malignant) ground truths were preserved. This limits our ability to evaluate subtype-specific performance and potential spectrum bias in the external cohort. Finally, our current evaluation primarily focused on discriminative metrics (AUC, MCC) and clinical net benefit (DCA). Future work should explore automated semantic feature extraction via Natural Language Processing (NLP) or the integration of genomic markers (27–31). Additionally, radiomic features present a promising quantitative complement to expert knowledge (32). However, overcoming challenges related to radiomic feature robustness and biological interpretability (33, 34) through standardized preprocessing and radiogenomic correlation will be crucial for developing reliable hybrid diagnostic systems (35–37).

## 6 Conclusion

In conclusion, this radiologist-assisted multimodal framework integrates non-contrast CT and radiologist-defined features for parotid tumor classification. Findings suggest fusion models outperform single-modal approaches by synergizing spatial and expert knowledge. While the cross-attention mechanism (AttenNet) did not show statistically significant quantitative superiority over simple concatenation, It may enhance qualitative interpretability by directing attention toward clinically relevant regions. While relying solely on non-contrast CT broadens clinical applicability, limitations-including small sample sizes, rare subtypes, and subjective manual features—require cautious interpretation. This proof-of-concept necessitates extensive multicenter validation, potentially incorporating radiomics and NLP, to conclusively establish clinical reliability.

## Data Availability

The raw data supporting the conclusions of this article will be made available by the authors, further inquiries can be directed to QY, 285990095@qq.com.
