# Supplementary material for: Radiologist-guided multimodal fusion network for differentiating parotid tumors on 3D non-contrast CT
Source: Front Oncol. 2026 Oct 15;16:1926379. doi: 10.3389/fonc.2026.1926379 (PMC13716596; doi:10.3389/fonc.2026.1926379)
Supplement: Supplementary file 1 [file Table1.docx]

**Inter-observer Agreement Among Three Radiologists for Visual-Semantic Features**

| **Visual-semantic features** | ****Fleiss' Kappa (*κ*)**** | ****95% CI**** | ****Level of Agreement**** |
| --- | --- | --- | --- |
| Tumor morphology | 0.82 | (0.78, 0.86) | Almost Perfect |
| Tumor margin | 0.85 | (0.81, 0.89) | Almost Perfect |
| Tumor necrosis | 0.74 | (0.69, 0.79) | Substantial |
| CT attenuation | 0.79 | (0.75, 0.83) | Substantial |

**Supplementary Table S1-1** *****κ*****, Fleiss' kappa coefficient; CI, confidence interval. The level of agreement based on *****κ***** values was interpreted as follows: 0.00–0.20, slight agreement; 0.21–0.40 fair agreement; 0.41–0.60, moderate agreement; 0.61–0.80, substantial agreement; and 0.81–1.00, almost perfect agreement.

| **Visual-semantic features** | **Radiologist A（s/case）** | **Radiologist B（s/case）** | **Radiologist C（s/case）** |
| --- | --- | --- | --- |
| Tumor morphology | 14.2 | 13.1 | 15.1 |
| Tumor margin | 18.3 | 17.2 | 19.7 |
| Tumor necrosis | 10.6 | 9.8 | 11.8 |
| CT attenuation | 19.4 | 18.5 | 20.4 |

**Supplementary Table S1-2** Values are presented as mean annotation time per case. The overall mean was calculated across the three experts and four semantic features.

**Time required for semantic feature extraction**
