# Supplementary material for: Radiologist-guided multimodal fusion network for differentiating parotid tumors on 3D non-contrast CT
Source: Front Oncol. 2026 Oct 15;16:1926379. doi: 10.3389/fonc.2026.1926379 (PMC13716596; doi:10.3389/fonc.2026.1926379)
Supplement: Supplementary file 2 [file Table2.docx]

| **Metric** | **3D-EfficientNet** | **3D-ResNet50** | **AttenNet(3D-EfficientNet)** | **AttenNet(3D-ResNet50)** | **Logistic** | **RF** | **SC(3D-EfficientNet)** | **SC(3D-ResNet50)** | **XGBoost** |
| --- | --- | --- | --- | --- | --- | --- | --- | --- | --- |
| **AUC** | 0.6640 [0.6002, 0.7249] | 0.7408 [0.7384, 0.8391] | 0.8903 [0.8494, 0.9264] | **0.9082** [0.8725, 0.9396] | 0.7864 [0.7317, 0.8326] | 0.8126 [0.7608, 0.8578] | 0.9035 [0.8696, 0.9340] | 0.8751 [0.8348, 0.9127] | 0.8207 [0.7712, 0.8633] |
| **F1score** | 0.5096 [0.4315, 0.5846] | 0.6232 [0.5492, 0.6901] | 0.7529 [0.6860, 0.8148] | 0.7695 [0.7064, 0.8245] | 0.6681 [0.5926, 0.7345] | 0.6681 [0.5926, 0.7345] | 0.7712 [0.7083, 0.8297] | 0.7525 [0.6888, 0.8115] | 0.6712 [0.5978, 0.7359] |
| **MCC** | 0.2693 [0.1695, 0.3670] | 0.4558 [0.3658, 0.5441] | 0.6498 [0.5628, 0.7343] | 0.6771 [0.5977, 0.7499] | 0.5237 [0.4279, 0.6147] | 0.5237 [0.4279, 0.6147] | 0.6776 [0.5968, 0.7550] | 0.6508 [0.5696, 0.7287] | 0.5286 [0.4340, 0.6180] |
| **PRAUC** | 0.3992 [0.3139, 0.4912] | 0.5322 [0.4337, 0.6422] | 0.7799 [0.6964, 0.8511] | 0.7750 [0.6843, 0.8570] | 0.4801 [0.3950, 0.5683] | 0.5229 [0.4319, 0.6177] | 0.7469 [0.6498, 0.8329] | 0.6361 [0.5300, 0.7386] | 0.5663 [0.4642, 0.6738] |
| **Precision** | 0.4120 [0.3333, 0.4904] | 0.5062 [0.4268, 0.5854] | 0.6870 [0.5969, 0.7669] | 0.6752 [0.5932, 0.7538] | 0.5903 [0.5000, 0.6767] | 0.5903 [0.5000, 0.6767] | 0.7035 [0.6209, 0.7850] | 0.6668 [0.5846, 0.7477] | 0.5951 [0.5076, 0.6800] |
| **Sensitivity** | 0.6711 [0.5794, 0.7596] | 0.8139 [0.7292, 0.8901] | 0.8350 [0.7582, 0.9109] | 0.8967 [0.8316, 0.9512] | 0.7723 [0.6867, 0.8491] | 0.7723 [0.6867, 0.8491] | 0.8554 [0.7810, 0.9223] | 0.8655 [0.7921, 0.9293] | 0.7723 [0.6867, 0.8491] |
| **Specificity** | 0.6277 [0.5663, 0.6860] | 0.6915 [0.6352, 0.7478] | 0.8506 [0.8025, 0.8945] | 0.8323 [0.7845, 0.8760] | 0.7895 [0.7362, 0.8408] | 0.7895 [0.7362, 0.8408] | 0.8599 [0.8154, 0.9016] | 0.8319 [0.7851, 0.8776] | 0.7937 [0.7410, 0.8442] |

**Supplementary Table S2**

**Performance comparison of different models for parotid tumor differentiation on the test set.**

**Supplementary Table S2** Data are presented as the mean value with 95% confidence intervals in brackets [95% CI]. **3D-EfficientNet** and **3D-ResNet50** represent baseline deep learning backbones. **AttenNet** denotes the proposed radiologist-guided attention fusion network. **SC** refers to the Score-level fusion (ensemble) strategy. **Logistic**, **RF** (Random Forest), and **XGBoost** represent traditional machine learning classifiers based on radiomics features.
