# Supplementary material for: Radiologist-guided multimodal fusion network for differentiating parotid tumors on 3D non-contrast CT
Source: Front Oncol. 2026 Oct 15;16:1926379. doi: 10.3389/fonc.2026.1926379 (PMC13716596; doi:10.3389/fonc.2026.1926379)
Supplement: Supplementary file 3 [file Table3.docx]

| **Comparison** | **b** | **c** | ***p*-value** | **Cohen's κ** |
| --- | --- | --- | --- | --- |
| **AttenNet(3D-ResNet)**  **vs**  **Radiologist A** | 3 | 2 | 1.0000 | 0.5000 |
| **AttenNet(3D-ResNet)**  **vs**  **Radiologist B** | 3 | 3 | 1.0000 | 0.4000 |
| **Radiologist A**  **vs**  **Radiologist B** | 2 | 3 | 1.0000 | 0.4898 |

**Supplementary Table S3** This table compares the standalone performance of your model against the two experts.

Notes:

- **b**: Number of cases where Method 1 was correct and Method 2 was incorrect.
- **c**: Number of cases where Method 1 was incorrect and Method 2 was correct.
- ***p*-value:** Calculated using the binomial exact distribution. A *p*-value > 0.05 indicates no statistically significant difference in diagnostic performance between the two methods.
- **Cohen's κ:** Represents the strength of agreement. Values between 0.41 and 0.60 are generally considered "Moderate Agreement."
