# Supplementary material for: Radiologist-guided multimodal fusion network for differentiating parotid tumors on 3D non-contrast CT
Source: Front Oncol. 2026 Oct 15;16:1926379. doi: 10.3389/fonc.2026.1926379 (PMC13716596; doi:10.3389/fonc.2026.1926379)
Supplement: Supplementary file 4 [file Table4.docx]

Supplementary Table S4 Spatial Overlap Consistency of ROI Annotations among Three Experts (N=367)

| Pairwise Comparison | N | Dice Similarity Coefficient | 95% CI |
| --- | --- | --- | --- |
| Expert A vs. Expert B | 367 | 0.89 ± 0.04 | [0.88, 0.90] |
| Expert B vs. Expert C | 367 | 0.86 ± 0.06 | [0.85, 0.87] |
| Expert A vs. Expert C | 367 | 0.88 ± 0.05 | [0.87, 0.89] |
| Overall Average | 367 | 0.877 ± 0.05 | **[0.86, 0.89]** |

The overall average Dice Similarity Coefficient (DSC) was calculated by **pooling the pairwise DSC values** (Expert A vs. B, B vs. C, and A vs. C) across all 367 cases, providing a single metric for the total inter-observer consensus.
