# Supplementary material for: Radiologist-guided multimodal fusion network for differentiating parotid tumors on 3D non-contrast CT
Source: Front Oncol. 2026 Oct 15;16:1926379. doi: 10.3389/fonc.2026.1926379 (PMC13716596; doi:10.3389/fonc.2026.1926379)
Supplement: Supplementary file 5 [file Table5.docx]

|  | Type | Center A | Center B | Center C | Total |
| --- | --- | --- | --- | --- | --- |
| Benign Tumors |  |  |  |  |  |
|  | Pleomorphic Adenoma | 112 | 5 | 3 | 120 |
|  | Warthin's Tumor | 61 | 8 | 5 | 74 |
|  | Basal Cell Adenoma | 31 | 9 | 0 | 40 |
|  | Other Benign Tumors | 23 | 1 | 2 | 26 |
| Malignant Tumors |  |  |  |  |  |
|  | Adenocarcinoma | 47 | 0 | 6 | 53 |
|  | Mucoepidermoid Carcinoma | 5 | 4 | 0 | 9 |
|  | Squamous Cell Carcinoma | 23 | 4 | 3 | 30 |
|  | Other Malignant Tumors | 12 | 2 | 1 | 15 |

**Supplementary Table S5**

Distribution of benign and malignant tumors across three hospitals (A, B, and C). The table summarizes the number of cases for each tumor type, including pleomorphic adenoma, Warthin's tumor, basal cell adenoma, and other benign tumors, as well as adenocarcinoma, mucoepidermoid carcinoma, squamous cell carcinoma, and other malignant tumors. Total counts for benign and malignant tumors are 260 and 107, respectively. Differences in the distribution across hospitals are noted.
