# Supplementary material for: Radiologist-guided multimodal fusion network for differentiating parotid tumors on 3D non-contrast CT
Source: Front Oncol. 2026 Oct 15;16:1926379. doi: 10.3389/fonc.2026.1926379 (PMC13716596; doi:10.3389/fonc.2026.1926379)
Supplement: Supplementary file 6 [file Table6.docx]

**Supplementary Table S6. Tumor characteristics and pathological distribution stratified by segmentation quality (Dice coefficient groups)**

| **Characteristics** | **High Dice (>0.85)** **(n = 200)** | **Intermediate Dice (0.70–0.85)** **(n = 120)** | **Low Dice (<0.70)** **(n = 27)** | **P value** |
| --- | --- | --- | --- | --- |
| **Maximum diameter (mm)** |  |  |  | **0.055*** |
| Mean ± SD | 44.55 ± 14.81 | 40.64 ± 14.53 | 38.19 ± 19.71 |  |
| Median (IQR) | 43.89 (34.31–52.77) | 39.28 (29.93–49.98) | 41.14 (26.46–53.90) |  |
| **Volume (cm³)** |  |  |  | **<0.001*** |
| Mean ± SD | 25.41 ± 25.29 | 16.16 ± 17.48 | 12.88 ± 12.34 |  |
| Median (IQR) | 17.82 (9.91–30.67) | 10.33 (5.34–21.31) | 9.47 (3.28–22.07) |  |
| **Dice coefficient** |  |  |  | **<0.001*** |
| Mean ± SD | 0.897 ± 0.030 | 0.794 ± 0.039 | 0.513 ± 0.221 |  |
| **Pathological class, n (%)** |  |  |  | **0.165†** |
| Benign | 150 (75.0%) | 79 (65.8%) | 21 (77.8%) |  |
| Malignant | 50 (25.0%) | 41 (34.2%) | 6 (22.2%) |  |

*Note: Data are presented as mean ± standard deviation (SD), median (interquartile range, IQR), or number of patients (with percentages).*

** P values were calculated using the Kruskal–Wallis H test for continuous variables with non-normal distributions.*

*† P values were calculated using the Pearson Chi-square test for categorical variables.*
