# Supplementary material for: Radiologist-guided multimodal fusion network for differentiating parotid tumors on 3D non-contrast CT
Source: Front Oncol. 2026 Oct 15;16:1926379. doi: 10.3389/fonc.2026.1926379 (PMC13716596; doi:10.3389/fonc.2026.1926379)
Supplement: Supplementary file 7 [file Table7.docx]

**Supplementary Table S7 Ablation analysis of attention and semantic features**.

| Baseline | Condition | AUC（95%CI） | Adjust p-value |
| --- | --- | --- | --- |
| 3D-ResNet | AttenNet | **0.9082** (0.8725,0.9390) | <0.01 |
|  | Attention output set to zero | 0.5360（0.4701,0.6042） |  |
|  | Semantic features set to zero | 0.5243 (0.4562,0.5891) |  |
| 3D-EfficientNet | AttenNet | **0.8903** (0.8494,0.9262) | <0.01 |
|  | Attention output set to zero | 0.5023 (0.4312,0.5683) |  |
|  | Semantic features set to zero | 0.5176 (0.4312,0.5683) |  |

#Values are presented as AUC with 95% confidence intervals. Setting the attention output or semantic features to zero was used to assess their contribution to model performance.
